# Advancing Insights into Biomarkers in Congenital Anomalies of the Kidney and Urinary Tract: A Scoping Review

**DOI:** 10.3390/cells15121083

**Published:** 2026-06-15

**Authors:** Francesco Maria Rosanio, Giulia Borgia, Elena Ferone, Adriano Braile, Seyedeh Fatemeh Hosseininasab, Mariantonia Braile

**Affiliations:** 1 Pediatric Emergency Department, Santobono-Pausilipon Children’s Hospital, 80129 Naples, Italy; 2 Department of Translational Medical Science, Pediatric Section, University of Federico II, 80131 Naples, Italy; 3 Department of Medical and Surgical Specialties and Dentistry, University of Campania “Luigi Vanvitelli”, 81100 Naples, Italy; 4 Department of Clinical Sciences and Translational Medicine, University of Tor Vergata, 00133 Rome, Italy; 5 Department of Molecular Medicine and Medical Biotechnologies, University of Naples Federico, 80145 Naples, Italy; 6 UOSD Laboratorio Territoriale SS Annunziata, ASL Napoli 1, 80145 Naples, Italy

**Keywords:** biomarkers, CAKUT (congenital anomalies of the kidney and urinary tract), nephrology, renal disease, urinary tract malformations

## Abstract

Background: Congenital anomalies of the kidney and urinary tract (CAKUT) comprise a heterogeneous spectrum of developmental disorders and represent the leading cause of chronic kidney disease and end-stage renal disease in the pediatric population. Although imaging remains the cornerstone of diagnosis, its limited ability to accurately assess disease severity and predict long-term outcomes has driven growing interest in urinary, serum, and tissue biomarkers as potential indicators of early renal injury. Objectives: To systematically summarize the current evidence on diagnostic and prognostic biomarkers in pediatric CAKUT, with particular focus on their potential clinical utility in early detection of renal injury and disease monitoring. Methods: A scoping review was conducted in accordance with PRISMA guidelines. PubMed, Embase, and Scopus were searched up to March 2026 using combinations of CAKUT-related terms and “biomarkers.” Studies involving human subjects with CAKUT that evaluated the diagnostic, prognostic, or therapeutic utility of biomarkers were included. Results: Out of 1130 records identified, 101 studies met the inclusion criteria. Urine was the most commonly analyzed biological sample. The principal biomarkers identified included NGAL, KIM-1, MCP-1, TGF-β1, CA19-9, β2-microglobulin, cystatin C, and microRNAs. Across various CAKUT subtypes—including posterior urethral valves, ureteropelvic junction obstruction, vesicoureteral reflux, and multicystic dysplastic kidney—these biomarkers showed significant associations with renal function, inflammatory activity, and fibrotic processes. Several biomarkers, particularly urinary NGAL, MCP-1, and CA19-9, demonstrated good diagnostic performance in differentiating obstructive from non-obstructive hydronephrosis and in predicting renal impairment. However, substantial heterogeneity in study design, along with the lack of standardized cutoff values, limits their translation into routine clinical practice. Conclusions: Current evidence underscores the potential of several biomarkers for the diagnosis and monitoring of CAKUT-related renal injury. Nevertheless, well-designed multicenter prospective studies are needed to validate their clinical utility and to support the integration of biomarker-based approaches with imaging in pediatric practice.

## 1. Introduction

Congenital anomalies of the kidney and urinary tract (CAKUT) comprise a broad and heterogeneous group of developmental disorders arising from disruptions in the tightly regulated processes of nephrogenesis and urinary tract morphogenesis during embryonic life. These anomalies affect the kidneys, ureters, bladder, and urethra, and include a wide spectrum of structural and functional defects such as renal agenesis, hypodysplasia, ureteropelvic junction obstruction (UPJO), vesicoureteral reflux (VUR), posterior urethral valves (PUV), and other forms of urinary tract obstruction (UTO) [1]. The phenotypic variability is substantial, ranging from mild abnormalities with spontaneous resolution to severe malformations associated with perinatal mortality or progressive renal failure. Increasing evidence supports a multifactorial etiology involving genetic variants—including copy number variations (CNVs)—as well as environmental influences, with a significant proportion of cases occurring in syndromic contexts [2]. CAKUT represent the leading cause of chronic kidney disease (CKD) and end-stage kidney disease (ESKD) in the pediatric population, accounting for up to 40–60% of cases in children and adolescents across multiple registries [3]. The clinical trajectory is highly variable and not fully predictable based on anatomical findings alone. While some patients maintain stable renal function over time, others develop progressive renal injury driven by maladaptive mechanisms such as hyperfiltration, tubular stress, inflammation, and fibrosis [3].

Currently, the diagnostic workup of CAKUT relies predominantly on imaging modalities. Prenatal and postnatal ultrasonography (US) is the cornerstone for initial detection, while voiding cystourethrography (VCUG), renal scintigraphy, and magnetic resonance imaging are used to further characterize anatomical and functional abnormalities [4].

However, imaging findings often show limited correlation with histopathological damage or long-term renal outcomes. For example, hydronephrosis (HN) detected on ultrasound may resolve spontaneously, whereas patients with minimal imaging abnormalities may still develop progressive renal dysfunction. Similarly, conventional laboratory parameters such as serum creatinine (sCr), estimated glomerular filtration rate (eGFR), blood urea nitrogen (BUN), and cystatin C (CysC) are relatively late indicators of kidney injury and may fail to detect early tubular damage or subclinical disease progression [5].

In this context, there has been increasing interest in the identification of non-invasive biomarkers capable of reflecting early pathophysiological changes in CAKUT. Among the most extensively studied are neutrophil gelatinase-associated lipocalin (NGAL) and kidney injury molecule-1 (KIM-1), which have demonstrated diagnostic utility across multiple CAKUT phenotypes—including hydronephrosis, UPJO, VUR, and PUV—and, in some studies, prognostic value for renal function decline and renal scarring [6].

Inflammatory and fibrotic pathways are also prominently represented. Biomarkers such as monocyte chemoattractant protein-1 (MCP-1), transforming growth factor-beta 1 (TGF-β1), interleukins (e.g., IL-6, IL-8, IL-1β), and tumor necrosis factor-alpha (TNF-α) have been associated with both diagnostic and prognostic aspects of CAKUT, particularly in obstructive uropathies and reflux nephropathy [3]. TGF-β1 and MCP-1, in particular, have shown relevance across multiple CAKUT phenotypes, suggesting a central role in fibrogenesis and progressive renal injury. In addition to protein biomarkers, emerging evidence highlights the role of microRNAs (miRNAs) as regulators of gene expression in kidney development and injury. Specific miRNAs—including miR-21-5p, miR-144, and others—have been implicated in CAKUT phenotypes such as hydronephrosis and urethral obstruction, suggesting potential utility as both diagnostic and mechanistic biomarkers [3]. Similarly, components of the renin–angiotensin system, such as ACE2 and angiotensin-(1–7), have been explored in PUV, further supporting the relevance of molecular pathways in disease characterization [3].

A broad spectrum of additional biomarkers reflects the complexity of CAKUT pathophysiology. These include tubular enzymes (e.g., N-acetyl-β-D-glucosaminidase [NAG], gamma-glutamyl transferase [GGT]), low-molecular-weight proteins (e.g., β2-microglobulin), and markers of extracellular matrix remodeling such as matrix metalloproteinases (MMPs) and their inhibitors (TIMPs), which are particularly relevant in obstructive nephropathies [3]. Moreover, novel candidates identified through proteomic and metabolomic approaches—including differentially expressed proteins (DEPs), metabolic signatures, and molecules such as fetuin-A and MMP-7—have expanded the landscape of potential biomarkers and may offer improved diagnostic and prognostic performance [3].

Given these considerations, a comprehensive synthesis of the available evidence is needed. Therefore, this scoping review aims to systematically map and summarize current knowledge on biomarkers in pediatric CAKUT, focusing on their diagnostic and prognostic roles across different phenotypes and their potential integration into clinical practice.

Moreover, this scoping review critically evaluates the clinical utility of biomarkers across CAKUT phenotypes, comparing their diagnostic and prognostic performance with conventional imaging, assessing specificity, validation status, and translational applicability.

## 2. Materials and Methods

### 2.1. Information Sources and Search Strategy

A scoping review was undertaken to comprehensively identify studies investigating the relationship between CAKUT in pediatric populations and biomarkers. A systematic literature search was performed across three electronic databases: Embase, PubMed, and Scopus. Search terms were combined using different Boolean strategies tailored to each database and included: CAKUT OR “congenital anomalies of the kidney and urinary tract” OR “kidney agenesis” OR “renal agenesis” OR “vesicoureteral reflux” OR “multicystic dysplastic kidney” OR “megaureter” OR “ureteropelvic junction obstruction” OR “posterior urethral valves” OR “kidney hypoplasia” OR “renal hypoplasia” OR “kidney dysplasia” OR “renal dysplasia” OR “renal ectopia” OR “kidney ectopia” OR “crossed fused renal ectopia” OR “horseshoe kidney” OR “hydronephrosis” OR “duplex kidney” OR “urethral strictures,” combined with the term “biomarkers.” Boolean operators (AND, OR) and Title/Abstract filters were applied to refine the search strategy. The final search was completed on March 2026. For reproducibility, a representative PubMed search string was: ((“congenital anomalies of the kidney and urinary tract” OR CAKUT OR “hydronephrosis” OR “ureteropelvic junction obstruction” OR “vesicoureteral reflux” OR “posterior urethral valves”) AND (biomarker OR biomarkers)), with appropriate adaptations for Embase and Scopus. Complete search strategies for all databases are available in Supplementary File S1. The PICO (Population, Intervention, Comparison, Outcomes) framework guided study eligibility [7].

The Population (P) included pediatric patients (neonates, infants, toddlers, and children) diagnosed with CAKUT or specific CAKUT subtypes (e.g., kidney agenesis, vesicoureteral reflux, hydronephrosis, multicystic dysplastic kidney, posterior urethral valves), as well as adults with a history of congenital kidney anomalies diagnosed in childhood. The Intervention (I) comprised biomarkers associated with CAKUT, including serum, urinary, and genetic biomarkers potentially reflecting disease presence, severity, or progression, as well as studies integrating imaging modalities (e.g., ultrasound, MRI, CT) with biomarker assessment. The Comparison (C) involved the evaluation of different biomarkers in terms of diagnostic performance, including sensitivity, specificity, and predictive value. The Outcomes (O) of interest included diagnostic accuracy, disease monitoring, response to treatment, and long-term prognosis.

The scope and reporting of this review followed the Preferred Reporting Items for Systematic Reviews and Meta-Analyses extension for Scoping Reviews (PRISMA-ScR) guidelines [8]. The PRISMA-ScR checklist is provided in Supplementary File S1.

### 2.2. Eligibility Criteria

The literature search was conducted by one author using predefined keyword combinations across the three databases. Retrieved records were merged into a single dataset, and duplicates were removed. Subsequently, three reviewers independently screened all remaining studies based on predefined inclusion criteria: (i) studies involving patients with CAKUT; (ii) studies evaluating biomarkers; and (iii) studies conducted on human subjects. Studies were excluded based on the following criteria: (i) titles or abstracts lacking at least one CAKUT-related term and at least one biomarker-related term; (ii) conference abstracts; (iii) editorials, letters, books, notes, or surveys; (iv) articles not pertinent to the review topic; (v) non-English publications; (vi) review articles (narrative reviews, systematic reviews, or meta-analyses); (vii) studies involving non-human subjects; and (viii) non-open access publications. Reasons for exclusion are detailed in Supplementary File S2. Titles and abstracts were independently assessed by three reviewers. Any discrepancies between reviewers were resolved through discussion. We limited our review to studies with accessible full texts (predominantly open access) to enable comprehensive data extraction and methodological appraisal; this choice reflected resource constraints rather than bias toward study outcomes.

### 2.3. Data Extraction and Quality Assessment

Following study selection, data were extracted through manual curation. The authors independently collected, verified, and synthesized relevant information from each included study to ensure consistency and reduce bias. Studies were included if they involved CAKUT populations and evaluated urinary, serum, genetic, or molecular biomarkers. Particular attention was given to obstructive uropathies, including posterior urethral valves, ureteropelvic junction obstruction (UPJO), and hydronephrosis. In addition, studies investigating inflammatory and fibrotic pathways in both upper and lower urinary tract disease were specifically analyzed, with emphasis on biomarkers implicated in tissue injury, immune activation, and extracellular matrix remodeling.

## 3. Results

### 3.1. Literature Research

**Figure 1:**
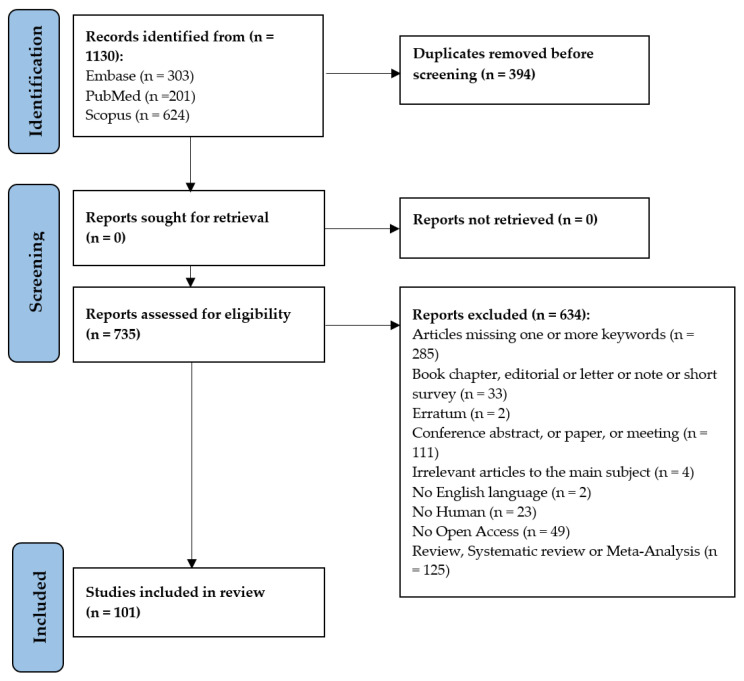
PRISMA-ScR flow-diagram showing research strategy [8].

The study selection process is outlined in the flow diagram (Figure 1), which summarizes the literature search strategy. A total of 1130 records were retrieved from PubMed, Embase, and Scopus using combinations of the terms “CAKUT” or “congenital anomalies of the kidney and urinary tract” and “biomarkers.” After removal of duplicate entries, 735 articles remained and were screened according to the predefined eligibility criteria. A total of 101 studies were included, encompassing prenatal, postnatal, and adult uropathies. Evidence was derived from fetal studies on urinary obstruction and inflammation, obstructive nephropathy models, and large biomarker validation cohorts.

### 3.2. Study Characteristics

The included studies were published between 1999 and 2024 (Table 1, Supplementary File S3). Sample sizes ranged from 1 to 287 participants, for a total of 7664 individuals across all studies. A variety of study designs were represented, including 42 case–control studies, 26 cohort studies (both prospective and retrospective), 8 interventional studies, 24 cross-sectional, observational, comparative, or clinical studies, and 1 case report. With regard to biological samples, urine was the most frequently analyzed specimen, reported in 66 studies, including fetal urine, bladder urine, renal pelvic urine, and midstream urine samples. Blood samples—such as serum, peripheral blood, fetal blood, and cord blood—were examined in 26 studies. Tissue specimens, including renal and ureteral tissue as well as biopsy samples, were analyzed in 6 studies, while amniotic fluid was investigated in 3 studies.

### 3.3. Biomarkers in Pediatric CAKUT

A total of 101 studies investigating biomarkers in pediatric CAKUT were included, covering a wide spectrum of disease phenotypes, including hydronephrosis (HN), ureteropelvic junction obstruction (UPJO), vesicoureteral reflux (VUR), posterior urethral valves (PUV), multicystic dysplastic kidney (MCDK), and other urinary tract obstructions (UTO). Across studies, biomarkers were primarily evaluated for their ability to (i) improve diagnostic discrimination, particularly between obstructive and non-obstructive conditions, (ii) detect early renal injury before decline in conventional parameters, and (iii) predict clinically relevant outcomes such as renal scarring, functional deterioration, and need for surgical intervention (Table 2) [9,10,11,12,13,14,15,16,17,18,19,20,21,22,23,24,25,26,27,28,29,30,31,32,33,34,35,36,37,38,39,40,41,42,43,44,45,46,47,48,49,50,51,52,53,54].

**Figure 2:**
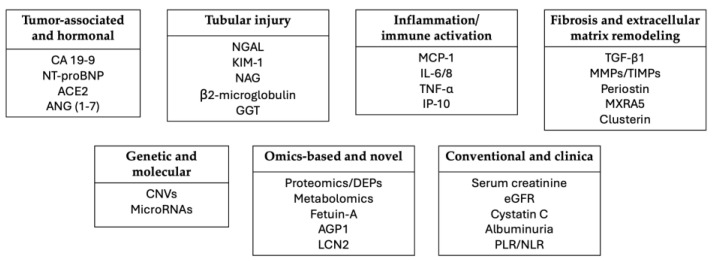
Mechanistic pathways linking biomarkers to tubular injury, inflammation, fibrosis, and metabolic/genetic alterations in CAKUT. Abbreviations: CAKUT, congenital anomalies of the kidney and urinary tract; CA 19-9, carbohydrate antigen 19-9; NT-proBNP, N-terminal pro-B-type natriuretic peptide; ACE2, angiotensin-converting enzyme 2; NGAL, neutrophil gelatinase-associated lipocalin; KIM-1, kidney injury molecule-1; NAG, N-acetyl-β-D-glucosaminidase; uGGT, urinary gamma-glutamyl transferase; B2M, beta-2-microglobulin; MCP-1, monocyte chemoattractant protein-1; IL-6, interleukin-6; IL-8, interleukin-8; TNF-α, tumor necrosis factor-α; CRP, C-reactive protein; TGF-β1, transforming growth factor-β1; ECM, extracellular matrix; MMPs, matrix metalloproteinases; TIMPs, tissue inhibitors of metalloproteinases; MXRA5, matrix-remodeling-associated protein 5; CNVs, copy number variants; miRNAs, microRNAs; DEPs, differentially expressed proteins; LCN2, lipocalin-2; BUN, blood urea nitrogen; eGFR, estimated glomerular filtration rate; ESR, erythrocyte sedimentation rate; PLR, platelet-to-lymphocyte ratio; NLR, neutrophil-to-lymphocyte ratio.

The biomarkers identified across pediatric CAKUT phenotypes can be grouped into distinct but interconnected pathophysiological categories, including tubular injury, inflammation and immune activation, fibrosis and extracellular matrix remodeling, genetic and molecular alterations, omics-based pathways, and conventional clinical markers. Although several biomarkers participate in overlapping biological processes, this classification highlights the principal mechanisms underlying renal injury progression and provides a structured framework for interpreting their potential diagnostic and prognostic applications. Figure 2 summarizes the major biomarker categories involved in CAKUT and their association with kidney damage pathways.

#### 3.3.1. Tumor-Associated and Hormonal Biomarkers

Carbohydrate antigen 19-9 (CA 19-9) was consistently reported as elevated in children with hydronephrosis and obstructive uropathies, with levels correlating with the degree of pelvic dilation and urinary stasis, suggesting that it reflects increased epithelial secretion and tubular stress under conditions of obstruction [9,10,11,12]. From a clinical standpoint, this biomarker appears particularly useful in differentiating obstructive from non-obstructive hydronephrosis, a common and often challenging scenario in pediatric urology, where imaging findings alone may be inconclusive [12]. Importantly, CA 19-9 levels have been shown to decrease following surgical correction, supporting its potential role as a marker of treatment response and resolution of obstruction [11,13]. In patients with PUV, persistently elevated CA 19-9 levels after valve ablation were associated with residual bladder dysfunction and ongoing renal impairment, indicating a possible prognostic role in identifying patients at risk for poor outcomes [19,29,48]. ACE2 and Ang-(1–7) were explored in PUV, where alterations in their levels reflected dysregulation of the renin–angiotensin system, a pathway known to contribute to renal fibrosis and progression of kidney disease [49]. Clinically, these markers may help identify patients with early activation of maladaptive pathways even before structural damage becomes evident, although their use remains investigational [35]. NT-proBNP was found to be elevated in hydronephrosis and UPJO, with levels correlating with the severity of obstruction and renal pelvic pressure [22,35]. This is clinically relevant as it suggests a link between renal obstruction and systemic hemodynamic stress, potentially offering an additional dimension beyond local imaging findings. Furthermore, higher NT-proBNP levels were associated with poorer renal outcomes and persistence of obstruction, indicating its potential role as a prognostic biomarker in clinical follow-up [22,35].

#### 3.3.2. Tubular Injury Biomarkers

NGAL emerged as one of the most robust and consistently validated biomarkers across all CAKUT phenotypes. Elevated urinary NGAL levels were observed in patients with hydronephrosis, UPJO, and VUR, even in the presence of normal serum creatinine and preserved eGFR, indicating its sensitivity for early tubular injury [16,17].

Clinically, this is highly relevant because it addresses a major limitation in current practice, where renal damage is often detected only at advanced stages. Several studies demonstrated that NGAL levels were significantly higher in obstructive versus non-obstructive hydronephrosis, suggesting its utility in guiding clinical decision-making, particularly in borderline cases where the indication for surgery is uncertain [16,21,36]. Moreover, NGAL levels decreased after surgical correction, indicating reversibility of tubular injury and supporting its role as a dynamic biomarker for monitoring therapeutic response [16]. From a prognostic perspective, elevated NGAL levels were associated with renal scarring and long-term functional impairment, particularly in VUR and obstructive uropathies, suggesting that it may help identify patients at higher risk for CKD progression [17,50,51]. KIM-1 demonstrated a similar but complementary profile, reflecting proximal tubular epithelial injury. Increased urinary KIM-1 levels were consistently reported in UPJO, VUR, and PUV, supporting its diagnostic value across different CAKUT phenotypes [15,16,18]. Clinically, KIM-1 appears particularly useful in identifying ongoing tubular damage even in clinically stable patients, thereby enabling earlier intervention. Importantly, KIM-1 levels were higher in obstructive conditions and correlated with severity of obstruction, suggesting a role in differentiating surgical from conservative cases [16,52]. Prognostically, elevated KIM-1 levels were associated with persistent renal dysfunction and suboptimal recovery after intervention, particularly in PUV, highlighting its value in long-term follow-up [15,16]. Other tubular markers, including NAG, GGT, AKP, and microglobulins, were frequently elevated in obstructive and reflux nephropathies, reflecting generalized tubular dysfunction [28,36,37]. Although less specific than NGAL and KIM-1, these markers may provide additional information in a multimarker approach, especially in settings where advanced biomarkers are not available.

#### 3.3.3. Inflammatory and Immune-Related Biomarkers

Inflammatory biomarkers were consistently associated with disease severity and progression across CAKUT phenotypes. MCP-1, a key chemokine involved in monocyte recruitment, was elevated in UPJO, PUV, and VUR, correlating with both obstruction severity and degree of renal injury [20,21]. Clinically, elevated MCP-1 levels may identify patients with active inflammatory processes who are at increased risk of fibrosis and progressive renal damage [14,16]. TGF-β1 emerged as a central mediator of fibrosis, with consistently elevated levels in patients with hydronephrosis, UPJO, PUV, and VUR [21,22,23]. From a clinical perspective, TGF-β1 is particularly relevant because it reflects irreversible fibrotic remodeling, which is a key determinant of long-term renal outcome. Higher TGF-β1 levels were associated with renal scarring and worse functional outcomes, suggesting its potential role as a prognostic biomarker [24,25]. Interleukins such as IL-6 and IL-8 were elevated in multiple CAKUT phenotypes, reflecting ongoing inflammatory activation [30,31,53]. Clinically, higher cytokine levels were associated with more severe disease and increased risk of renal scarring, particularly in VUR [32]. TNF-α further supported the central role of inflammation in CAKUT progression, with elevated levels correlating with both structural damage and functional decline [30,41].

#### 3.3.4. Fibrosis and Extracellular Matrix Remodeling

Markers of extracellular matrix remodeling provided insight into structural kidney damage. MMPs and TIMPs were significantly altered in UPJO and hydronephrosis, reflecting active remodeling processes [40]. Clinically, imbalance between MMPs and TIMPs may indicate progressive fibrosis and identify patients at risk of irreversible damage. Collagen fragments and MXRA5 further reflected fibrotic remodeling and were associated with more advanced disease stages [54]. Periostin and clusterin were associated with epithelial injury and extracellular matrix deposition, particularly in congenital obstruction [42,55].

#### 3.3.5. Genetic and Molecular Biomarkers

Genetic alterations, particularly CNVs, were identified in a subset of patients and were associated with more severe and syndromic CAKUT phenotypes [43]. Clinically, this supports the integration of genetic testing in selected cases, especially when multiple anomalies or atypical presentations are present. MicroRNAs showed differential expression patterns in hydronephrosis and urethral obstruction, suggesting a role in regulating key pathways involved in kidney injury [33]. Although not yet used in clinical practice, these markers may represent future tools for early diagnosis and targeted therapy.

#### 3.3.6. Omics-Based and Novel Biomarkers

Proteomic and metabolomic approaches identified novel biomarkers associated with CAKUT, including DEPs, amino acid profiles, and metabolic signatures [44,45,46]. These findings suggest that metabolic dysregulation and mitochondrial dysfunction may play a role in disease progression. Specific biomarkers such as fetuin-A, AGP1, and LCN2 were associated with renal stress and inflammation, indicating potential roles in early diagnosis and monitoring [47].

#### 3.3.7. Conventional and Clinical Biomarkers

Traditional biomarkers such as serum creatinine, cystatin C, BUN, and eGFR remained essential in clinical practice but were shown to have limited sensitivity for early kidney injury [26,27]. Clinically, these markers are more useful for monitoring established disease rather than early detection. Urinary markers such as albumin and IgG were associated with glomerular damage, while α1-microglobulin reflected tubular dysfunction, particularly in VUR [39]. Inflammatory indices such as CRP, ESR, and hematological ratios (PLR, NLR) were associated with disease severity but lacked specificity, limiting their standalone clinical utility [30,38].

### 3.4. Diagnostic Accuracy and Clinical Performance of Urinary Biomarkers

Table 3 summarizes the diagnostic and prognostic performance of selected urinary biomarkers investigated in pediatric CAKUT. Only biomarkers with available quantitative diagnostic accuracy data, including cut-off values, sensitivity, specificity, and/or AUC, were included in the analysis. Biomarkers lacking standardized or directly comparable performance metrics across studies are discussed separately in the text. Among the investigated biomarkers, CA 19-9 demonstrated one of the highest diagnostic accuracies for differentiating obstructive from non-obstructive hydronephrosis, with a reported cut-off of 95 U/mL, sensitivity of 95%, specificity of 96%, and AUC of 0.99 [6]. Similarly, NGAL emerged as a sensitive marker of early tubular injury, with a urinary cut-off of 20.57 ng/mg creatinine achieving 82% sensitivity and 100% specificity (AUC 0.923) [15], although its diagnostic performance varied substantially across studies. KIM-1 showed moderate but clinically relevant diagnostic accuracy, with reported sensitivity and specificity values of 92.3% and 83.3%, respectively [16]. In addition, urinary MCP-1/Cr, ET-1/Cr, and NAG/Cr ratios were significantly higher in neonates requiring pyeloplasty, suggesting a potential role in stratifying obstruction severity [20]. However, substantial heterogeneity exists across studies in terms of assay methodology, urine normalization strategies, cohort characteristics, and follow-up duration, limiting direct comparability between reported thresholds. Most currently available evidence derives from relatively small single-center pediatric cohorts, and only a limited number of biomarkers have undergone external or multicenter validation. Consequently, despite promising diagnostic and prognostic performance, the integration of these biomarkers into routine pediatric urological practice remains limited and requires further prospective validation studies.

### 3.5. Phenotype-Specific Evidence

#### 3.5.1. Hydronephrosis and Ureteropelvic Junction Obstruction (UPJO)

Hydronephrosis and ureteropelvic junction obstruction (UPJO) represent the most extensively investigated CAKUT phenotypes in biomarker research. Among the available candidates, CA 19-9, NGAL, and KIM-1 demonstrated the strongest associations with obstruction severity, tubular injury, and risk of functional deterioration.

CA 19-9 consistently showed high diagnostic accuracy in differentiating obstructive from non-obstructive hydronephrosis, with urinary levels correlating with pelvic dilation, urinary stasis, and severity of obstruction [6]. Importantly, CA 19-9 concentrations frequently decreased after pyeloplasty, supporting its potential role as a dynamic biomarker of treatment response [9].

NGAL emerged as one of the most sensitive markers of early tubular injury in UPJO, with elevated urinary levels observed even in patients with preserved serum creatinine and eGFR [14,15]. Higher NGAL concentrations were associated with severe obstruction and poorer renal functional parameters, suggesting potential utility in identifying borderline cases requiring surgical intervention [14,15]. Similarly, KIM-1 reflected proximal tubular epithelial injury and correlated with obstruction severity and postoperative renal recovery [15,16].

Inflammatory and fibrosis-related biomarkers, including MCP-1, TGF-β1, MMPs, and TIMPs, were also associated with progressive renal remodeling and worsening hydronephrosis severity [19,23,40,56]. However, despite promising diagnostic and prognostic performance, substantial heterogeneity in assay methodology, urine normalization strategies, and reported cut-off values currently limits routine clinical implementation [18].

#### 3.5.2. Vesicoureteral Reflux (VUR)

In VUR, biomarkers associated with tubular injury, inflammation, and renal scarring appear to have the greatest clinical relevance. NGAL and KIM-1 were the biomarkers most consistently associated with reflux severity, recurrent urinary tract infections, and progressive renal dysfunction [15,17,51]. Elevated urinary NGAL levels correlated with renal scarring and functional decline, even in patients with preserved serum creatinine, supporting its role as an early marker of subclinical tubular injury [17,51]. KIM-1 similarly reflected ongoing proximal tubular epithelial damage and was associated with chronic nephropathy and reduced renal reserve [15,16]. Higher concentrations of both biomarkers were reported in children with obstructive nephropathy and reflux-related renal injury compared with controls and were associated with increased risk of permanent renal scarring [17,51].

Inflammatory biomarkers also contributed to risk stratification in VUR. MCP-1, IL-6, IL-8, and TNF-α were associated with higher grades of reflux, inflammatory activation, and increased risk of renal fibrosis [19,29,31,32,53]. Nevertheless, despite encouraging associations, variability in study design, assay techniques, and threshold values currently limits their incorporation into routine clinical algorithm [18,57].

#### 3.5.3. Posterior Urethral Valves (PUV)

In PUV, the biomarkers most strongly associated with disease severity and long-term renal outcome included CA 19-9, NT-proBNP, and markers related to renin–angiotensin system activation. Elevated urinary CA 19-9 levels correlated with obstruction severity and frequently remained increased after valve ablation in patients with persistent bladder dysfunction and ongoing renal impairment, suggesting a potential prognostic role [10].

Alterations in ACE2 and Ang-(1–7) reflected dysregulation of antifibrotic and renoprotective pathways, supporting the hypothesis that early activation of the renin–angiotensin system contributes to progressive fibrosis and CKD development in PUV [49]. In addition, NT-proBNP emerged as a potential marker of systemic hemodynamic stress secondary to urinary obstruction, with higher levels correlating with renal pelvic pressure and poorer renal outcomes [22,35].

Tubular injury biomarkers, including NGAL and KIM-1, were also elevated in children with PUV and persistent renal dysfunction, indicating ongoing epithelial injury despite surgical correction [23,42]. However, most currently available evidence derives from relatively small exploratory cohorts, and prospective multicenter validation studies remain lacking [18,57].

#### 3.5.4. Multicystic Dysplastic Kidney (MCDK) and Other Rare CAKUT Phenotypes

Evidence regarding biomarkers in multicystic dysplastic kidney (MCDK) and other rare CAKUT phenotypes remains limited. Current data mainly derive from exploratory proteomic, metabolomic, and microRNA-based studies investigating molecular signatures associated with abnormal nephrogenesis and dysplastic remodeling [33,34,52]. Candidate biomarkers, including urinary microRNAs, differentially expressed proteins (DEPs), amino acid profiles, and metabolic signatures, have shown potential associations with disease severity and renal dysplasia [34,45,47,52]. Additional biomarkers such as fetuin-A, AGP1, and LCN2 were linked to renal stress and inflammatory activation [47]. However, no biomarker has yet demonstrated reproducible diagnostic performance, validated cut-off values, or sufficient external validation for clinical application. Consequently, these biomarkers remain investigational and are not currently integrated into routine diagnostic or prognostic pathways [18,57,58,59].

## 4. Discussion

The present review highlights both the promise and the current limitations of biomarker research in pediatric CAKUT. Although the literature has expanded substantially over the last two decades, the field remains highly heterogeneous, encompassing different phenotypes, biological matrices, study designs, and outcome definitions [1,2,3]. Rather than representing a single disease entity, CAKUT comprises a spectrum of disorders characterized by partially overlapping but biologically distinct mechanisms of renal injury. Consequently, biomarkers should not be interpreted as universally applicable tools, but rather in relation to specific phenotypes and underlying pathophysiological pathways.

A major finding emerging from this review is the progressive convergence of evidence toward a limited number of interconnected biological processes, particularly tubular injury, inflammation, fibrosis, and extracellular matrix remodeling. Across different cohorts and CAKUT subtypes, biomarkers such as NGAL and KIM-1 consistently reflected early tubular epithelial damage, whereas MCP-1, IL-6, and TGF-β1 were more closely associated with inflammatory activation and fibrotic progression [14,15,19,23,31,32,40,41,53]. This mechanistic clustering reinforces the concept that progression toward CKD in CAKUT is largely mediated by chronic tubular stress and maladaptive remodeling rather than by isolated anatomical abnormalities alone.

Importantly, the clinical significance of biomarkers differed substantially according to phenotype. In hydronephrosis and UPJO, CA 19-9, NGAL, and KIM-1 demonstrated the most reproducible diagnostic performance [6,15,16]. Among these, CA 19-9 showed the highest specificity for distinguishing obstructive from non-obstructive hydronephrosis and was one of the few biomarkers to demonstrate relatively reproducible cut-offs across independent studies [6,9]. In addition, the reduction in CA 19-9 levels after pyeloplasty suggests that this marker may reflect dynamic changes in obstruction severity rather than static anatomical findings alone [9]. From a translational perspective, this is particularly relevant because one of the major unresolved clinical problems in pediatric urology remains the identification of patients with asymptomatic hydronephrosis who may benefit from early surgical intervention rather than prolonged observation.

NGAL and KIM-1, by contrast, appear to function primarily as markers of subclinical tubular injury. Both biomarkers were elevated even in patients with preserved serum creatinine and eGFR, suggesting greater sensitivity for early renal damage compared with conventional laboratory markers [14,15,17]. However, despite encouraging diagnostic performance, their specificity remains imperfect because both markers may also increase in other inflammatory or ischemic renal conditions. Consequently, their principal value may lie less in isolated diagnostic discrimination and more in risk stratification when integrated with imaging and functional assessment.

In VUR, the biological interpretation of biomarkers appears substantially different. Here, biomarkers were generally less useful for diagnosing reflux itself and more relevant for identifying patients at risk of reflux nephropathy, recurrent inflammatory injury, and renal scarring. NGAL and KIM-1 were consistently associated with chronic tubular injury and progressive renal dysfunction, while MCP-1, IL-6, and TGF-β1 reflected persistent inflammatory and profibrotic activation [14,16,19,31,32,53]. These findings suggest that, in VUR, biomarker panels may have greater prognostic than diagnostic utility. Nevertheless, many inflammatory biomarkers lack disease specificity because they are influenced by urinary tract infection, systemic inflammation, and procedural stress, limiting their standalone clinical applicability.

In PUV, biomarker research increasingly supports the concept that ongoing renal damage may persist despite anatomical correction of obstruction. CA 19-9 retained both diagnostic and prognostic relevance, whereas biomarkers associated with renin–angiotensin system activation and systemic hemodynamic stress—including ACE2, Ang-(1–7), and NT-proBNP—provided preliminary mechanistic insights into maladaptive remodeling and progression toward CKD [10,35,49]. However, most studies investigating these pathways remain exploratory and are based on relatively small cohorts without external validation. Similarly, evidence regarding MCDK and other rare CAKUT phenotypes remains limited and largely restricted to experimental proteomic, metabolomic, and microRNA-based studies [33,34,44,45,46,47,52].

An important translational issue raised by the current literature concerns the actual bedside applicability of available biomarkers. Although many markers demonstrated statistically significant associations with obstruction severity, fibrosis, renal scarring, or functional decline, very few have reached the level of validation required to modify clinical management. At present, imaging modalities—including ultrasonography, VCUG, scintigraphy, and MRI—remain the cornerstone of diagnosis and follow-up [4]. Biomarkers may improve risk stratification when combined with imaging, but none currently provides sufficient evidence to independently guide clinical “grey-zone” decisions, such as conservative versus surgical management of asymptomatic hydronephrosis. This gap between biological plausibility and clinical implementation remains one of the major unresolved challenges in the field.

Another major limitation concerns methodological heterogeneity. Substantial variability exists in assay techniques, urine normalization strategies, timing of sampling, age distribution, hydration status, and outcome definitions [57,59,60]. These differences significantly limit reproducibility and complicate comparison between studies. In particular, standardized cut-off values remain unavailable for most biomarkers, including NGAL, KIM-1, MCP-1, and fibrosis-related mediators [57,59,60]. Moreover, the majority of studies are retrospective, single-center investigations involving relatively small pediatric populations, with only limited multicenter validation. These limitations reduce generalizability and partly explain why most biomarkers remain confined to research settings.

The increasing use of omics technologies—including transcriptomics, proteomics, metabolomics, and extracellular vesicle analysis—represents one of the most innovative aspects of recent CAKUT research. Early studies identified differentially expressed proteins, metabolic signatures, and microRNA profiles associated with obstruction severity and renal dysfunction [45,46,47].

Nevertheless, these approaches remain largely exploratory and currently lack standardization, reproducibility, and longitudinal validation. At present, omics-based biomarkers should therefore be considered hypothesis-generating rather than clinically actionable tools. However, their integration with conventional biomarkers and imaging may ultimately facilitate more refined phenotype stratification and precision medicine approaches.

Overall, the available evidence suggests that the future role of biomarkers in CAKUT will likely depend on multimarker and phenotype-oriented strategies rather than on single-marker approaches. CA 19-9 appears particularly promising for obstructive phenotypes, whereas NGAL and KIM-1 may provide earlier detection of tubular injury and progressive renal dysfunction. Inflammatory and fibrosis-related markers appear more useful for prognostic stratification than for diagnosis. Future prospective multicenter studies integrating biomarkers with imaging, clinical phenotyping, and longitudinal renal outcomes will be essential to determine whether these molecular tools can meaningfully improve management and long-term prognosis in pediatric CAKUT.

## 5. Conclusions

Current evidence indicates that biomarkers in pediatric CAKUT primarily reflect three interconnected biological processes: tubular injury, inflammation, and fibrotic remodeling. However, their clinical utility appears strongly phenotype-dependent rather than universally applicable across all CAKUT subtypes. Among the available candidates, CA 19-9, NGAL, and KIM-1 currently demonstrate the most reproducible associations with obstructive nephropathy, tubular injury, and risk of renal deterioration, particularly in hydronephrosis and UPJO.

Despite promising diagnostic and prognostic performance, no biomarker has yet achieved sufficient validation to replace imaging or independently guide clinical decision-making. Important limitations—including assay heterogeneity, lack of standardized cut-offs, small pediatric cohorts, and limited multicenter validation—continue to hinder clinical translation. Moreover, many inflammatory and fibrosis-related biomarkers show limited specificity because they reflect downstream responses shared across multiple renal and systemic conditions.

Emerging omics-based approaches, including proteomics, metabolomics, transcriptomics, and extracellular vesicle analysis, may improve future phenotype stratification and precision medicine strategies, although these technologies remain largely exploratory. Future research should prioritize prospective multicenter validation studies, harmonization of analytical methodologies, development of age-adjusted reference ranges, and integration of biomarkers with imaging and clinical parameters in order to achieve clinically meaningful implementation in pediatric nephrology.

## Figures and Tables

**Table 1:** Characteristics of Included Studies and Analyzed Biological Samples.

Section	Category	Details	Number of Studies
**General Information**	Publication period	1999–2024	-
	Total participants	7664	-
	Sample size (range)	1–287 participants	-
**Study Designs**	Case–control	-	42
	Cohort (prospective & retrospective)	-	26
	Interventional	-	8
	Cross-sectional/observational comparative/clinical	-	24
	Case report	-	1
**Biological Samples**	Urine	Fetal, bladder, renal pelvic, midstream	66
	Blood	Serum, peripheral, fetal, cord blood	26
	Tissue	Renal, ureteral, biopsy samples	6
	Amniotic fluid	-	3

**Table 2:** Biomarkers in Pediatric CAKUT: Diagnostic and Prognostic Evidence.

Biomarker	CAKUT Subtype	Diagnostic Role	Prognostic Role	
**CA 19-9**	HN, UTO, PUV	Identifies obstruction (HN, UTO, PUV)	Predicts outcome in UTO and PUV	[6,9,10,11,12,13]
**NGAL**	HN, UPJO, VUR, UTO, PUV, MCDK	Early marker of renal injury and obstruction	Predicts renal impairment and scarring	[14,15,16,17]
**KIM-1**	HN, UPJO, VUR, UTO, PUV, MCDK	Tubular injury marker	Associated with disease severity and progression	[15,16,18]
**MCP-1**	HN, UPJO, VUR, PUV, MCDK	Marker of inflammation	Predicts progression and fibrosis	[14,16,19,20,21]
**TGF-β1**	HN, UPJO, VUR, PUV	Fibrosis marker	Associated with disease progression and scarring	[21,22,23,24,25]
**Cystatin C (CysC)**	CAKUT, HN, UPJO, PUV	Renal function assessment	Predicts CKD progression	[5,16,17,23,26,27]
**Serum creatinine (sCr)**	CAKUT, HN, VUR, UTO	Standard renal marker	Predicts long-term renal function	[5,26]
**β2-microglobulin**	HN, UPJO, VUR, PUV, UTO	Tubular dysfunction marker	Associated with renal damage	[17,28]
**IL-6**	PUV, UPJO, VUR	Inflammatory marker	Predicts severity in PUV and VUR	[14,29,30,31,32]
**IL-8**	VUR	Marker of inflammation	Associated with disease progression	[31,32]
**NGAL-related miRNAs**	HN	Molecular markers of injury	Under investigation	[33]
**Other miRNAs**	CAKUT, HN, urethral stricture	Diagnostic potential	Not yet established	[33,34]
**NT-proBNP**	HN, UPJO	Reflects pressure/obstruction	May predict outcomes	[22,35]
**NAG**	HN, UPJO, VUR, PUV	Tubular enzyme marker	Limited prognostic data	[20,36,37]
**CRP/hematologic markers**	VUR, urethral stricture	Inflammatory indicators	Associated with severity	[30,38]
**Albumin/µALB/RBP**	VUR, UTO	Glomerular/tubular damage	Predict renal injury	[29,39]
**MMPs/TIMPs**	HN, UPJO	ECM remodeling markers	Associated with fibrosis	[40]
**TNF-α**	PUV, UPJO	Inflammatory cytokine	Linked to progression	[29,30,41]
**EGF**	VUR, PUV, UPJO	Renal repair marker	Potential prognostic role	[42]
**Genetic variants (CNVs)**	CAKUT	Etiological role	May predict phenotype	[2,43]
**Metabolites/amino acids**	PUV, UPJO	Emerging biomarkers	Under investigation	[44,45,46,47]

Abbreviation: CA 19-9 (Carbohydrate Antigen 19-9); CAKUT (Congenital Anomalies of the Kidney and Urinary Tract); CKD (Chronic Kidney Disease); CNVs (Copy Number Variants); CRP (C-reactive Protein); CysC (Cystatin C); EGF (Epidermal Growth Factor); HN (Hydronephrosis); IL-6 (Interleukin-6); IL-8 (Interleukin-8); KIM-1 (Kidney Injury Molecule-1); MCDK (Multicystic Dysplastic Kidney); MCP-1 (Monocyte Chemoattractant Protein-1); MMPs (Matrix Metalloproteinases); NAG (N-acetyl-β-D-glucosaminidase); NGAL (Neutrophil Gelatinase-Associated Lipocalin); NT-proBNP (N-terminal pro-B-type Natriuretic Peptide); PUV (Posterior Urethral Valves); RBP (Retinol-Binding Protein); sCr (Serum Creatinine); TGF-β1 (Transforming Growth Factor Beta 1); TIMPs (Tissue Inhibitors of Metalloproteinases); TNF-α (Tumor Necrosis Factor Alpha); UPJO (Ureteropelvic Junction Obstruction); UTO (Urinary Tract Obstruction); VUR (Vesicoureteral Reflux); µALB (Microalbuminuria).

**Table 3:** Diagnostic and prognostic performance of selected urinary biomarkers in pediatric CAKUT.

Biomarker	Phenotype	Cut-Off (Unit)	Sensitivity (%)	Specificity (%)	AUC	Reference	Comments/Clinical Notes
CA 19-9	UPJO/HN/PUV	95 U/mL	95	96	0.99	[6,12]	High diagnostic accuracy for obstructive hydronephrosis
NGAL	UPJO/HN/VUR	20.57 ng/mg Cr	82	100	0.923	[14,15]	Early marker of tubular injury
NGAL	UPJO	18.74 pg/mL	79	34	0.607	[14,15]	Moderate diagnostic performance
KIM-1	UPJO	0.687 ng/mg Cr	92.3	83.3	0.89	[15,16]	Good diagnostic performance
KIM-1	UPJO	466.06 pg/mL	75	38	0.61	[15,16]	Moderate performance
MCP-1 (MCP-1/Cr)	HN/UPJO	0.927 ng/mg	77	72	0.732	[20]	Inflammatory marker for obstruction severity
ET-1 (ET-1/Cr)	HN/UPJO	0.5709 ng/mg	75	67	–	[20]	Vasoactive marker, limited validation
NAG (NAG/Cr)	HN/UPJO	1.1913 U/mg	62	67	–	[37]	Tubular enzyme marker, limited data

Abbreviations: CAKUT, congenital anomalies of the kidney and urinary tract; UPJO, ureteropelvic junction obstruction; HN, hydronephrosis; VUR, vesicoureteral reflux; PUV, posterior urethral valves; NGAL, neutrophil gelatinase-associated lipocalin; KIM-1, kidney injury molecule-1; MCP-1, monocyte chemoattractant protein-1; ET-1, endothelin-1; NAG, N-acetyl-β-D-glucosaminidase; Cr, creatinine; AUC, area under the receiver operating characteristic curve.

## Data Availability

The original contributions presented in this study are included in the article/Supplementary Materials. Further inquiries can be directed to the corresponding author.

## Supplementary Materials

The following supporting information can be downloaded at: https://www.mdpi.com/article/10.3390/cells15121083/s1. Supplementary File S1. PRISMA Checklist. Supplementary File S2. List of studies not included and exclusion reasons. Supplementary File S3. List of included studies.

## Abbreviations

β2-MG	β2-microglobulina
CA 19-9	Carbohydrate Antigen 19-9
CAKUT	Congenital Anomalies of the Kidney and Urinary Tract
CKD	Chronic Kidney Disease
CNVs	Copy Number Variants
CRP	C-reactive Protein
CysC	Cystatin C
ECM	Extracellular Matrix
EGF	Epidermal Growth Factor
ESKD	End-Stage Kidney Disease
HN	Hydronephrosis
IL-6	Interleukin-6
IL-8	Interleukin-8
KIM-1	Kidney Injury Molecule-1
L-FABP	Liver-type Fatty Acid-Binding Protein
MCDK	Multicystic Dysplastic Kidney
MCP-1	Monocyte Chemoattractant Protein-1
miRNA	microRNA
MMPs	Matrix Metalloproteinases
NAG	N-acetyl-β-D-glucosaminidase
NGAL	Neutrophil Gelatinase-Associated Lipocalin
NT-proBNP	N-terminal pro-B-type Natriuretic Peptide
PICO	Population, Intervention, Comparison, Outcomes
PUV	Posterior Urethral Valves
RBP	Retinol-Binding Protein
sCr	Serum Creatinine
TGF-β1	Transforming Growth Factor Beta 1
TIMPs	Tissue Inhibitors of Metalloproteinases
TNF-α	Tumor Necrosis Factor Alpha
UPJO	Ureteropelvic Junction Obstruction
US	Ultrasound
UTO	Urinary Tract Obstruction
VCUG	Voiding Cystourethrography
VUR	Vesicoureteral Reflux
µALB	Microalbuminuria
