# Supplementary material for: Advancing Insights into Biomarkers in Congenital Anomalies of the Kidney and Urinary Tract: A Scoping Review
Source: Cells. 2026 Jun 15;15(12):1083. doi: 10.3390/cells15121083 (PMC13296763; doi:10.3390/cells15121083)
Supplement: Supplementary file 1 [file cells-15-01083-s001.zip › cells-4327446-supplementary.pdf]

Supplementary File S1

Preferred Reporting Items for Systematic reviews and Meta-Analyses extension for Scoping Reviews (PRISMA-ScR) Checklist

SECTION	ITEM	PRISMA-ScR CHECKLIST ITEM	REPORTED ON PAGE #
**TITLE**			
Title	1	Identify the report as a scoping review.	1
**ABSTRACT**			
Structured summary	2	Provide a structured summary that includes (as applicable): background, objectives, eligibility criteria, sources of evidence, charting methods, results, and conclusions that relate to the review questions and objectives.	1
**INTRODUCTION**			
Rationale	3	Describe the rationale for the review in the context of what is already known. Explain why the review questions/objectives lend themselves to a scoping review approach.	1-2
Objectives	4	Provide an explicit statement of the questions and objectives being addressed with reference to their key elements (e.g., population or participants, concepts, and context) or other relevant key elements used to conceptualize the review questions and/or objectives.	2
**METHODS**			
Protocol and registration	5	Indicate whether a review protocol exists; state if and where it can be accessed (e.g., a Web address); and if available, provide registration information, including the registration number.	3-4
Eligibility criteria	6	Specify characteristics of the sources of evidence used as eligibility criteria (e.g., years considered, language, and publication status), and provide a rationale.	3-4
Information sources*	7	Describe all information sources in the search (e.g., databases with dates of coverage and contact with authors to identify additional sources), as well as the date the most recent search was executed.	3-4
Search	8	Present the full electronic search strategy for at least 1 database, including any limits used, such that it could be repeated.	3-4
Selection of sources of evidence†	9	State the process for selecting sources of evidence (i.e., screening and eligibility) included in the scoping review.	3-4
Data charting process‡	10	Describe the methods of charting data from the included sources of evidence (e.g., calibrated forms or forms that have been tested by the team before their use, and whether data charting was done independently or in duplicate) and any processes for obtaining and confirming data from investigators.	3-4
Data items	11	List and define all variables for which data were sought and any assumptions and simplifications made.	3-4
Critical appraisal of individual sources of evidence§	12	If done, provide a rationale for conducting a critical appraisal of included sources of evidence; describe	3

St. Michael's
Inspired Care.
Inspiring Science.

SECTION	ITEM	PRISMA-ScR CHECKLIST ITEM	REPORTED ON PAGE #
		the methods used and how this information was used in any data synthesis (if appropriate).	
Synthesis of results	13	Describe the methods of handling and summarizing the data that were charted.	4
**RESULTS**			
Selection of sources of evidence	14	Give numbers of sources of evidence screened, assessed for eligibility, and included in the review, with reasons for exclusions at each stage, ideally using a flow diagram.	5
Characteristics of sources of evidence	15	For each source of evidence, present characteristics for which data were charted and provide the citations.	5-9
Critical appraisal within sources of evidence	16	If done, present data on critical appraisal of included sources of evidence (see item 12).	5-9
Results of individual sources of evidence	17	For each included source of evidence, present the relevant data that were charted that relate to the review questions and objectives.	6-9
Synthesis of results	18	Summarize and/or present the charting results as they relate to the review questions and objectives.	5-9
**DISCUSSION**			
Summary of evidence	19	Summarize the main results (including an overview of concepts, themes, and types of evidence available), link to the review questions and objectives, and consider the relevance to key groups.	10-11
Limitations	20	Discuss the limitations of the scoping review process.	10-11
Conclusions	21	Provide a general interpretation of the results with respect to the review questions and objectives, as well as potential implications and/or next steps.	10
**FUNDING**			
Funding	22	Describe sources of funding for the included sources of evidence, as well as sources of funding for the scoping review. Describe the role of the funders of the scoping review.	11

JBI = Joanna Briggs Institute; PRISMA-ScR = Preferred Reporting Items for Systematic reviews and Meta-Analyses extension for Scoping Reviews.

* Where *sources of evidence* (see second footnote) are compiled from, such as bibliographic databases, social media platforms, and Web sites.

† A more inclusive/heterogeneous term used to account for the different types of evidence or data sources (e.g., quantitative and/or qualitative research, expert opinion, and policy documents) that may be eligible in a scoping review as opposed to only studies. This is not to be confused with *information sources* (see first footnote).

‡ The frameworks by Arksey and O'Malley (6) and Levac and colleagues (7) and the JBI guidance (4, 5) refer to the process of data extraction in a scoping review as data charting.

§ The process of systematically examining research evidence to assess its validity, results, and relevance before using it to inform a decision. This term is used for items 12 and 19 instead of "risk of bias" (which is more applicable to systematic reviews of interventions) to include and acknowledge the various sources of evidence that may be used in a scoping review (e.g., quantitative and/or qualitative research, expert opinion, and policy document).

From: Tricco AC, Lillie E, Zarin W, O'Brien KK, Colquhoun H, Levac D, et al. PRISMA Extension for Scoping Reviews (PRISMAScR): Checklist and Explanation. Ann Intern Med. 2018;169:467–473. doi: 10.7326/M18-0850.

Supplmentary File S2. List of studies not included and exclusion reasons.

Authors		Title	Year	Exclusion Criteria
Abd El Gayed E	et al.	Evaluation of miRNA 130a-3P and miRNA 301a-3P in Egyptian patients with urinary bladder carcinoma	2024	Articles missing one or more keywords
Abdennadher W	et al.	Fetal urine biochemistry at 13-23 weeks of gestation in lower urinary tract obstruction: Criteria for in-utero treatment	2015	Articles missing one or more keywords
Abou Heidar N	et al.	Inflammatory serum markers predicting spontaneous ureteral stone passage	2020	Articles missing one or more keywords
AbuMaziad A	et al.	The role of novel COQ8B mutations in glomerulopathy and related kidney defects	2021	Articles missing one or more keywords
Acosta A	et al.	Primary Ureteral Thyroid Transcription Factor 1-Positive Small Cell Neuroendocrine Carcinoma	2015	Review
Aghaways I	et al.	Role of inflammatory markers in predicting spontaneous passage of ureteral stones less than 10 mm	2024	Articles missing one or more keywords
Akarken I	et al.	Is endothelial glycocalyx damage a cause of renal scarring in vesicoureteral reflux with febrile urinary tract infection?	2021	Articles missing one or more keywords
Akdemir G	et al.	Survival and renal outcome in fetuses with Lower Urinary Obstruction (LUTO) with and without intra-uterine vesicoamniotic shunting.A ten years experience of a cohort	2013	Conference Abstract
Albalat A	et al.	Clinical application of urinary proteomics/peptidomics	2011	Review
Alberti C	et al.	Congenital ureteropelvic junction obstruction: Physiopathology, decoupling of tout court pelvic dilatation-obstruction semantic connection, biomarkers to predict renal damage evolution	2012	Review
Al-Hussain T	et al.	Plasmacytoid acinar adenocarcinoma of the prostate: a newly described variant of prostate cancer	2019	Articles missing one or more keywords
Ali Q	et al.	De Novo Ulcerative Colitis in a Patient with Rapidly Progressive Glomerulonephritis (RPGN) after Renal Transplant	2024	Conference Abstract
Alizadeh F	et al.	Urinary carbohydrate antigen 19-9/creatinine ratio: A non-invasive marker for follow-up of unilateral ureteropelvic junction obstruction in children	2018	Articles missing one or more keywords
Allory Y	et al.	Bladder tumor histo-seminar – case 6: Invasive urothelial carcinoma, with inverted growth (UroA/FGFR3 molecular type)	2016	Articles missing one or more keywords
Al-Mashhadi A	et al.	Changes in arterial pressure and markers of nitric oxide homeostasis and oxidative stress following surgical correction of hydronephrosis in children	2018	Articles missing one or more keywords
Alnajar H	et al.	Tumor-immune microenvironment revealed by Imaging Mass Cytometry in a metastatic sarcomatoid urothelial carcinoma with a prolonged response to pembrolizumab	2022	Articles missing one or more keywords
Al-Sayyad A	et al.	Evaluation of biomarkers to differentiate upper from lower urinary tract infections in children	2011	Irrelevant
Ambarsari C	et al.	Urinary extracellular vesicles: Potential biomarkers for vesicoureteral reflux	2024	Book chapter

Amini E	et al.	The role of serum and urinary carbohydrate antigen 19-9 in predicting renal injury associated with ureteral stone	2016	Articles missing one or more keywords
Anandkumar D	et al.	Kidney injury molecule-1 is it a predictive marker for renal diseases?	2023	Articles missing one or more keywords
Ando M	et al.	Clinical Characteristics of Retroperitoneal Fibrosis Patients at a Tertiary Hospital in Japan	2023	Articles missing one or more keywords
Ansari M	et al.	Bladder contractility index in posterior urethral valve: A new marker for early prediction of progression to renal failure	2018	No Open Access
Aoyagi J	et al.	Trends of bone mineral density and bone quality in a paediatric kidney transplant recipient: A case report	2024	Articles missing one or more keywords
Archana P	et al.	Surrogate imaging markers of Urodynamic proven bladder dysfunction in posterior urethral valves: A comprehensive evaluation	2023	No Open Access
Arciuolo D	et al.	Postchemotherapy Endometrioid to Gastrointestinal Histotype Shift in Recurrent Endometrial Carcinoma	2022	Articles missing one or more keywords
Argani P	et al.	Ossifying Fibromyxoid Tumor of the Genitourinary Tract: Report of 4 Molecularly Confirmed Cases of a Diagnostic Pitfall	2023	Articles missing one or more keywords
Arlandis S	et al.	Think Tank 2: How Do We Precisely Define the “High Risk Bladder” and What Are the Interrelationships Between Inflammation, Blood Flow, Fibrosis, and Loss of Bladder Compliance?	2024	Review
Armean I	et al.	A rare case of urinary tract infection with Burkholderia cepacia in a male child	2018	Articles missing one or more keywords
Arnqvist H	et al.	Early increase in HbA1c trajectory predicts development of severe microangiopathy in patients with type 1 diabetes: The VISS study	2024	Articles missing one or more keywords
Assadi F	et al.	Urinary polyomavirus: novel biomarker of congenital ureteropelvic junction obstruction	2020	No Open Access
Avello A	et al.	Urine MMP7 as a kidney injury biomarker	2024	Review
Babu P	et al.	Inflammatory pseudotumor of kidney masquerading as renal carcinoma	2015	Articles missing one or more keywords
Babu R	et al.	Comparison of different pathological markers in predicting pyeloplasty outcomes in children	2020	Articles missing one or more keywords
Badawi A	et al.	Comparative profile for COVID-19 cases from China and North America: Clinical symptoms, comorbidities and disease biomarkers	2021	Articles missing one or more keywords
Badawy M	et al.	Prolonged fever and hyperferritinaemia: A puzzling diagnosis of neonatal herpes simplex virus infection during COVID-19 pandemic	2021	Articles missing one or more keywords
Bagchi P	et al.	Correction to: Significance of inflammatory biomarkers and urethral histology in patients with urethral stricture disease in relation to treatment outcome—a single centre prospective study in the north-eastern part of India (African Journal of Urology, (2022), 28, 1, (1), 10.1186/s12301-021-00252-9)	2022	Erratum
Bagińska J	et al.	Evaluation of Urinary L-FABP as a Tubular Damage Marker in Pediatric Neurogenic Bladder—A Pilot Study	2024	Articles missing one or more keywords

Baisakh M	et al.	Primary round cell sarcomas of the urinary bladder with EWSR1 rearrangement: a multi-institutional study of thirteen cases with a review of the literature	2020	Articles missing one or more keywords
Bajpai M	et al.	Congenital anomalies of the kidney and urinary tract, biomarkers, and chronic kidney disease in children: A trajectory for the surgeon-scientists of the next generation	2022	Editorial
Banerjee A	et al.	Role of different urinary biomarkers in the differentiation of ureteropelvic junction obstruction from transient hydronephrosis	2024	Conference Abstract
Barinotti A	et al.	Serum Biomarkers of Renal Fibrosis: A Systematic Review	2022	Review
Bartoli F	et al.	Prospective Study on Several Urinary Biomarkers as Indicators of Renal Damage in Children with CAKUT	2019	No Open Access
Bartucci R	et al.	Vanin 1: Its physiological function and role in diseases	2019	Review
Baryła M	et al.	Protein biomarkers in assessing kidney quality before transplantation-current status and future perspectives (Review)	2024	Review
Bashar K	et al.	Predictive parameters of arteriovenous fistula functional maturation in a population of patients with end-stage renal disease	2015	Articles missing one or more keywords
Basta J	et al.	A sall1-NuRD interaction regulates multipotent nephron progenitors and is required for loop of henle formation	2017	Articles missing one or more keywords
Batmaz S	et al.	Can we use neutrophil gelatinaseassociated lipocalin as a kidney damage marker in children with vesicoureteral reflux?	2012	Conference Abstract
Becerir T	et al.	Urinary excretion of pentraxin-3 correlates with the presence of renal scar following acute pyelonephritis in children	2019	Articles missing one or more keywords
Becknell B	et al.	Molecular Basis of Renal Adaptation in a Murine Model of Congenital Obstructive Nephropathy	2013	Articles missing one or more keywords
Beetz R	et al.	Congenital dilatation of the upper urinary tract: Current diagnostic and treatment concepts	2019	Articles missing one or more keywords
Beetz R	et al.	Treatment concepts for connatal uropathies	2010	Articles missing one or more keywords
Benamran D	et al.	Risk stratification for upper tract urinary carcinoma	2020	Review
Benz K	et al.	Urine mirna biomarkers that are differentially expressed in lichen sclerosus induced and non-lichen sclerosus induced urethral stricture disease	2021	Conference Abstract
Berton AM	et al.	Idiopathic Urethral Stricture and Nephrogenic Diabetes Insipidus: The Odd Couple	2019	Articles missing one or more keywords
Bhardwaj N	et al.	Primary seminal vesicle adenocarcinoma: A case report of rare entity and discussion of its differential diagnosis using immunohistochemical approach for the core biopsy specimen	2020	Articles missing one or more keywords
Bishop K	et al.	Nephrolithiasis	2020	Review
Bitaraf M	et al.	Upper Tract Urothelial Carcinoma (UTUC) Diagnosis and Risk Stratification: A Comprehensive Review	2023	Review

Bitsori M	et al.	Urine IL-8 concentrations in infectious and non-infectious urinary tract conditions	2011	Articles missing one or more keywords
Bolgeri M	et al.	Neutrophil gelatinase-associated lipocalin (NGAL) as a biomarker of renal injury in patients with ureteric stones: a pilot study	2021	Articles missing one or more keywords
Boulang CL	et al.	Metabolic Phenotyping: A Novel Technology Giving Improved Understanding in Renal Medicine	2017	Conference Abstract
Branco BC	et al.	Novel Biomarkers for Posterior Urethral Valve	2023	Review
Brandström P	et al.	Urinary Tract Infection in Children	2022	Review
Brawer MK	et al.	Best of the 2007 AUA Annual Meeting: Highlights from the 2007 Annual Meeting of the American Urological Association, May 19-24, 2007, Anaheim, CA	2007	Meeting
Breinbjerg A	et al.	Risk factors for kidney scarring and vesicoureteral reflux in 421 children after their first acute pyelonephritis, and appraisal of international guidelines	2021	Articles missing one or more keywords
Brewin A	et al.	The Use of Neutrophil Gelatinase–Associated Lipocalin (NGAL) as a Diagnostic and Prognostic Biomarker in Urinary Tract Obstruction: a Systematic Review	2022	Review
Brewin A	et al.	Role of urinary biomarkers for diagnosis and prognosis of kidney stone disease	2021	Review
Broeren M	et al.	Urine flow cytometry is an adequate screening tool for urinary tract infections in children	2019	Articles missing one or more keywords
Bruschi M	et al.	A new proteomic approach to the study of Peritoneal effluents in pediatric patients	2012	Conference Abstract
Bruschi M	et al.	The hidden message of peritoneal dialysis effluent in paediatric patients	2012	Conference Abstract
Bryniarski P	et al.	Influence of nephrectomy on solitary kidney function in children with non-malignant diseases	2009	Articles missing one or more keywords
Buchbinder D	et al.	When Screening for Severe Combined Immunodeficiency (SCID) with T Cell Receptor Excision Circles Is Not SCID: a Case-Based Review	2021	Articles missing one or more keywords
Buffin-Meyer B	et al.	The ANTENATAL multicentre study to predict postnatal renal outcome in fetuses with posterior urethral valves: Objectives and design	2020	Articles missing one or more keywords
Bukharina AB	et al.	Omics Technologies in Screening for Kidney Disease in Children with Congenital Uropathy	2022	Articles missing one or more keywords
Cachat F	et al.	Microalbuminuria and hyperfiltration in subjects with nephro-urological disorders	2013	Articles missing one or more keywords
Cai PY	et al.	Ureteropelvic Junction Obstruction/Hydronephrosis	2023	Articles missing one or more keywords
Cai X	et al.	Association between chinese visceral adiposity index and risk of kidney stones in a health screening population: an ultrasonography based cross-sectional study	2024	Articles missing one or more keywords
Cajaiba M	et al.	Congenital capillary proliferation of the kidney: a distinctive renal vascular lesion of childhood	2017	Articles missing one or more keywords

Çamlar S	et al.	The role of dynamic renal scintigraphy on clinical decision making in hydronephrotic children	2017	Articles missing one or more keywords
Campbell J	et al.	Biomarkers in Urethral Stricture Disease and Benign Lower Urinary Tract Disease	2023	Review
Cañadas-Garre M	et al.	Genetic susceptibility to chronic kidney disease - Some more pieces for the heritability puzzle	2019	Review
Capuano I	et al.	Parapelvic Cysts: An Imaging Marker of Kidney Disease Potentially Leading to the Diagnosis of Treatable Rare Genetic Disorders? A Narrative Review of the Literature	2022	Review
Caubet C	et al.	Advances in urinary proteome analysis and biomarker discovery in pediatric renal disease	2010	Review
Cayci F	et al.	Endocan levels in children with renal hypodysplasia	2021	Articles missing one or more keywords
Cerqueira DM	et al.	MicroRNAs in kidney development and disease	2022	Review
Cerrolaza JJ	et al.	Quantitative Ultrasound for Measuring Obstructive Severity in Children with Hydronephrosis	2016	No Open Access
Cerrolaza JJ	et al.	Hydronephrosis severity diagnosis from ultrasound imaging biomarkers	2014	Conference Abstract
Cetin N	et al.	Diagnostic Values of Immature Granulocytes Detected by the Sysmex XN 9000 Hematology Analyzer in Children with Urinary Tract Infections	2023	Articles missing one or more keywords
Chahin M	et al.	Triple-Negative Lobular Breast Cancer Causing Hydronephrosis	2020	Articles missing one or more keywords
Chaikof E	et al.	The Society for Vascular Surgery practice guidelines on the care of patients with an abdominal aortic aneurysm	2018	Articles missing one or more keywords
Chang TC	et al.	Positive predictive value of ct urography for upper tract urothelial carcinoma diagnosis using diagnostic ureteroscopy as the reference standard	2017	Conference Abstract
Chen H	et al.	Prognostic analysis of inflammatory response-related genes and biomarkers in patients with urothelial carcinoma of ureter	2023	Articles missing one or more keywords
Chen J	et al.	Urothelial carcinoma of the graft kidney with molecular analyses: a rare case report	2021	Articles missing one or more keywords
Chen J	et al.	Current status of artificial intelligence applications in urology and their potential to influence clinical practice	2019	Review
Chen PH	et al.	VALUE OF D-DIMER IN RISK STRATIFICATION FOR THROMBOEMBOLISM IN PATIENTS WITH ATRIAL FIBRILLATION AND LOW CHA2DS2-VASC SCORE	2022	Conference Abstract
Chen W	et al.	Pregnancy Zone Protein as an Emerging Biomarker for Cardiovascular Risk in Pediatric Chronic Kidney Disease	2023	Articles missing one or more keywords
Chevalier RL	et al.	CAKUT: A Pediatric and Evolutionary Perspective on the Leading Cause of CKD in Childhood	2023	Review
Chevalier RL	et al.	Prognostic factors and biomarkers of congenital obstructive nephropathy	2016	Review

Chevalier RL	et al.	Congenital Urinary Tract Obstruction: The Long View	2015	Review
Chevalier RL	et al.	Pathogenesis of renal injury in obstructive uropathy	2006	Review
Chevalier RL	et al.	Mechanisms of renal injury and progression of renal disease in congenital obstructive nephropathy	2010	Conference Paper
Chevalier RL	et al.	Chronic partial ureteral obstruction and the developing kidney	2008	Conference Paper
Chien TM	et al.	The predictive value of systemic immune-inflammation index on bladder recurrence on upper tract urothelial carcinoma outcomes after radical nephroureterectomy	2021	Articles missing one or more keywords
Chimenz R	et al.	Febrile Urinary Tract Infections in Children: The Role of High Mobility Group Box-1	2023	Articles missing one or more keywords
Ching CB	et al.	Urinary epidermal growth factor and monocyte chemotactic protein-1 as biomarkers of renal injury in patients with obstructed nephropathy	2022	Articles missing one or more keywords
Ching CB	et al.	Commentary to "Urinary Biomarkers Can Identify the Need for Pyeloplasty in Presence of Supranormal Differential Renal Function in Antenatally Diagnosed Unilateral Hydronephrosis"	2022	Note
Cho Y	et al.	The De Ritis (aspartate transaminase/alanine transaminase) ratio as a predictor of oncological outcomes in patients after surgery for upper urinary tract urothelial carcinoma	2017	Articles missing one or more keywords
Chu D	et al.	Diagnostic Accuracy of Point-of-care Nitrite and Leukocyte Esterase Dipstick Test for the Screening of Pediatric Urinary Tract Infections	2021	Articles missing one or more keywords
Chung H	et al.	Chronic exposure to ethylenethiourea induces kidney injury and polycystic kidney in mice	2019	No Human
Chung R	et al.	Genitourinary involvement of immunoglobulin G4-related disease	2021	Letter
Chung R	et al.	DURABILITY OF CLINICAL COMPLETE RESPONSE TO NAC FOR MIBC WITH ADVERSE PATHOLOGIC FEATURES	2024	Conference Abstract
Clemens M	et al.	Phase II, multicenter, open-label, randomized study of YM155 plus docetaxel as first-line treatment in patients with HER2-negative metastatic breast cancer	2015	Articles missing one or more keywords
Collins DE	et al.	Clinical assessment of urinary tract damage during sustained-release estrogen supplementation in mice	2017	Articles missing one or more keywords
Conconi D	et al.	Potential role of BCL2 in the recurrence of uterine smooth muscle tumors of uncertain malignant potential	2017	Articles missing one or more keywords
Corona F	et al.	Effect of the janus kinase inhibitor baricitinib in the treatment of COPA syndrome	2022	Conference Abstract
Corradini F	et al.	Comparative assessment of cultures from oral and urethral stem cells for urethral regeneration	2016	Review
Courville K	et al.	Chronic kidney disease of nontraditional causes in central Panama	2022	Articles missing one or more keywords
Crane C	et al.	RENAL AUTOTRANSPLANTATION IN AN ADOLESCENT BOY	2019	Conference Abstract

Crippa BL	et al.	Perinatal cystatin c as biomarker of nephron endowment	2020	Conference Abstract
Cui Y	et al.	Effective management of advanced colon cancer genotyping microsatellite stable/microsatellite instable-low with Kirsten rat sarcoma viral oncogene mutation using nivolumab plus ipilimumab combined with regorafenib and irinotecan: A case report	2021	Articles missing one or more keywords
D'Alessandro M	et al.	Fourier transform infrared analysis of urinary calculi and metabolic studies in a group of Sicilian children	2017	Articles missing one or more keywords
da Silva AJ	et al.	Pediatric chronic kidney disease: blood cell count indexes as inflammation markers	2023	Articles missing one or more keywords
Dai X	et al.	Urine Macrophages Polarization Predicts Renal Function Recovery after Nephron-Sparing Surgery in Patients with Renal Cell Carcinoma	2022	Articles missing one or more keywords
Dal Lago S	et al.	Torque Teno Virus: A Promising Biomarker in Kidney Transplant Recipients	2024	Articles missing one or more keywords
Danilova E	et al.	URINE METABOLOME IN THE DIAGNOSIS OF CHRONIC KIDNEY DISEASE	2023	Review
Dawman L	et al.	Copeptin as a potential biomarker of chronic kidney disease to predict the disease progression in children with chronic kidney disease	2024	Articles missing one or more keywords
de Ruiter B	et al.	Phase 1 Study of Chemoradiotherapy Combined with Nivolumab ± Ipilimumab for the Curative Treatment of Muscle-invasive Bladder Cancer	2022	Articles missing one or more keywords
Decramer S	et al.	Predicting the clinical outcome of congenital unilateral ureteropelvic junction obstruction in newborn by urinary proteome analysis	2006	Note
Decramer S	et al.	Urine in clinical proteomics	2008	Review
Delgado J	et al.	Renal diffusion tensor imaging (DTI): Are quantitative DTI values useful in the evaluation of children with ureteropelvic junction obstruction (UPJO)?	2017	Conference Abstract
Demidova K	et al.	Mathematical analysis of individual biomarker profiles of kidney damage in children with vesicoureteral reflux	2022	No English
Deryugina L	et al.	BIOMARKERS OF KIDNEY DAMAGE IN CHILDREN WITH CONGENITAL ANOMALIES OF THE KIDNEY AND URINARY TRACT (REVIEW OF LITERATURE)	2024	Review
Deshpande A	et al.	Current strategies to predict and manage sequelae of posterior urethral valves in children	2018	Articles missing one or more keywords
Desiderio C	et al.	Capillary electrophoresis-mass spectrometry: Recent trends in clinical proteomics	2010	Review
Diamantopoulos LN	et al.	Patient (pt) characteristics	2019	Conference Abstract
Dong Y	et al.	Research advances of carbohydrate antigen 19-9 as a biochemical marker of congenital hydrone- phrosis	2022	Articles missing one or more keywords
Dong Y	et al.	IGA NEPHROPATHY WITH NORMAL IGA AND COMPLEMENT LEVELS	2022	Conference Abstract

Dorsey HG	et al.	Mass-Spectrometry Analysis of Urinary Biomarkers of Endothelial Injury in Sickle Cell Anemia Patients	2020	Conference Abstract
Drannik GN	et al.	Immunologic and enzymologic urinary assessment of surgical efficacy in children with decompensated obstructed megaureter	2014	Conference Abstract
Duineveld C	et al.	Ex Vivo Test of Complement Dysregulation in Atypical Hemolytic Uremic Syndrome Kidney Transplant patients: A Pilot Study	2024	Articles missing one or more keywords
Dunlap J	et al.	TdT-positive infiltrate in inflamed pediatric kidney a potential diagnostic pitfall	2017	Conference Paper
Dușa C	et al.	A Multimodal Fuzzy Approach in Evaluating Pediatric Chronic Kidney Disease Using Kidney Biomarkers	2024	Articles missing one or more keywords
Eckes T	et al.	Consistent alteration of chain length-specific ceramides in human and mouse fibrotic kidneys	2021	Articles missing one or more keywords
Edwards-Richards A	et al.	Capillary rarefaction: An early marker of microvascular disease in young hemodialysis patients	2014	Articles missing one or more keywords
Ehrhardt-Humbert L	et al.	Angiotensin 1-7 as a novel biomarker of AKI in pediatric kidney transplant recipients	2020	Conference Abstract
El Atta H	et al.	Restricted diffusion MRI as a functional biomarker for the assessment of acute calcular upper urinary tract obstruction: initial experience	2021	Articles missing one or more keywords
El-Dydamony E	et al.	Novel Biomarkers for Posterior Urethral Valve	2023	Review
El-Zaatari Z	et al.	Pleomorphic giant cell carcinoma of prostate: Rare tumor with unique clinicopathological, immunohistochemical, and molecular features	2021	Review
Enevold Olsen N	et al.	Eosinophilic cystitis presenting as possible pediatric rhabdomyosarcoma in conventional imaging including18f-fdg-pet/ct/mri—a rare case	2021	Articles missing one or more keywords
Ergunay T	et al.	Extracellular vesicles in kidney development and pediatric kidney diseases	2024	Review
Etzion O	et al.	Liver involvement in turner's syndrome	2015	Conference Abstract
Evans M	et al.	TREC Screening for WHIM Syndrome	2021	Articles missing one or more keywords
Fahimi D	et al.	Clinical manifestations, laboratory markers, and renal ultrasonographic examinations in 1-month to 12-year-old Iranian children with pyelonephritis: a six-year cross-sectional retrospective study	2021	Articles missing one or more keywords
Fan X	et al.	Bladder-sparing approaches for muscle invasive bladder cancer: a narrative review of current evidence and future perspectives	2023	Review
Fang D	et al.	Comparison of clinicopathologic characteristics, epigenetic biomarkers and prognosis between renal pelvic and ureteral tumors in upper tract urothelial carcinoma	2018	Articles missing one or more keywords
Fang D	et al.	Homogenous or heterogenous biological behaviors between ureteral and renal pelvic tumors? a comparison of clinical features	2016	Conference Abstract
Fanos V	et al.	Metabolomics in neonatology: Fact or fiction?	2013	Review
Farrugia MK	et al.	Report on The Society for Fetal Urology panel discussion on the selection criteria and intervention for fetal bladder outlet obstruction	2017	Articles missing one or more keywords

Fàrster B	et al.	Validation of pre-treatment risk stratification parameters according to EAU Guidelines on upper tract urothelial carcinoma (UTUC)	2018	Conference Abstract
Fasoulakis Z	et al.	The Role of microRNAs Identified in the Amniotic Fluid	2020	Articles missing one or more keywords
Fathallah-Shaykh S	et al.	Progression of pediatric CKD of nonglomerular origin in the CKiD cohort	2015	Articles missing one or more keywords
Fazel M	et al.	Arginine Levels in Neonatal Hydronephrosis	2025	No Open Access
Fédou C	et al.	Comparison of the amniotic fluid and fetal urine peptidome for biomarker discovery in renal developmental disease	2020	Articles missing one or more keywords
Fidan C	et al.	Effects of hyperuricemia on renal function in pediatric renal transplant recipients	2015	Articles missing one or more keywords
Filip S	et al.	Urinary proteomics and molecular determinants of chronic kidney disease: Possible link to proteases	2014	Review
Foerster B	et al.	Validation of eau guideline's pretreatment risk stratification parameters in upper tract urothelial carcinoma (UTUC)	2018	Conference Abstract
Font A	et al.	Predictive signature of response to neoadjuvant chemotherapy in muscle-invasive bladder cancer integrating mRNA expression, taxonomic subtypes, and clinicopathological features	2023	Articles missing one or more keywords
Forster CS	et al.	Association between urodynamic parameters and urine neutrophil gelatinase–associated lipocalin concentrations in children with neuropathic bladders	2019	Articles missing one or more keywords
Forster CS	et al.	Urinary NGAL deficiency in recurrent urinary tract infections	2017	Articles missing one or more keywords
Forster CS	et al.	Neutrophil gelatinase-associated lipocalin: utility in urologic conditions	2017	Editorial
Fossum M	et al.	Editorial comment on urinary markers related to UPJO "A novel urinary biomarker protein panel to identify children with ureteropelvic junction obstruction–A pilot study"	2020	Note
Froehlich J	et al.	Urinary proteomics yield pathological insights for ureteropelvic junction obstruction	2016	Articles missing one or more keywords
Froehlich JW	et al.	Identification of potential urinary biomarkers of clinically significant ureteropelvic junction obstruction	2012	Conference Abstract
Fu F	et al.	Prenatal diagnosis of fetal multicystic dysplastic kidney via high-resolution whole-genome array	2016	Articles missing one or more keywords
Fu SQ	et al.	Research advances of biomarkers related to congenital hydronephrosis	2024	Review
Fujii Y	et al.	Distinct molecular subtypes and a high diagnostic urinary biomarker of upper urinary tract urothelial carcinoma	2020	Conference Abstract
Funt S	et al.	Neoadjuvant Atezolizumab With Gemcitabine and Cisplatin in Patients With Muscle-Invasive Bladder Cancer: A Multicenter, Single-Arm, Phase II Trial	2022	Articles missing one or more keywords
Gao J	et al.	Neoadjuvant PD-L1 plus CTLA-4 blockade in patients with cisplatin-ineligible operable high-risk urothelial carcinoma	2020	Articles missing one or more keywords

Gao J	et al.	A pilot presurgical study evaluating anti-PD-L1 durvalumab (durva) plus anti-CTLA-4 tremelimumab (treme) in patients (pts) with high-risk muscle-invasive bladder carcinoma (MIBC) who are ineligible for cisplatin-based neoadjuvant chemotherapy (NAC)	2019	Conference Abstract
Gartner V	et al.	Novel variants in SPTAN1 without epilepsy: An expansion of the phenotype	2018	Articles missing one or more keywords
Gavrilovici C	et al.	The Role of Urinary NGAL in the Management of Primary Vesicoureteral Reflux in Children	2023	Review
Geminiganesan S	et al.	UTILITY OF URINARY BIOMARKERS IN THE DIAGNOSIS AND PROGNOSIS OF CHILDREN WITH HYDRONEPHROSIS	2025	Conference Abstract
Gherasim CO	et al.	Updated immunohistochemistry characterization of well-differentiated neuroendocrine tumors of the kidney	2018	Conference Abstract
Gkalonaki I	et al.	Pathogenesis and prognosis of intrarenal reflux	2023	Articles missing one or more keywords
Göger YE	et al.	Can Urinary KIM-1 and NGAL Predict Management Endoscopic Surgery in Acute Unilateral Obstructive Stone Disease? Results from a Prospective Cohort Study	2022	Articles missing one or more keywords
González-Cuadrado C	et al.	Hemodialysis-Associated Immune Dysregulation in SARS-CoV-2-Infected End-Stage Renal Disease Patients	2023	Articles missing one or more keywords
Goswami S	et al.	Objectified kidney ultrasound echogenicity and size metrics as potential predictors for kidney function in children	2024	Articles missing one or more keywords
Goyal K	et al.	Hepcidin and proinflammatory markers in children with chronic kidney disease: A case-control study	2018	Articles missing one or more keywords
GPN	et al.	53rd Annual Meeting of the Society of Pediatric Nephrology	2022	Conference Review
Grach S	et al.	40-Year-Old Man With Left Leg Swelling and Abdominal Pain	2021	Short survey
Grashei M	et al.	Simultaneous magnetic resonance imaging of pH, perfusion and renal filtration using hyperpolarized 13C-labelled Z-OMPD	2023	Articles missing one or more keywords
Greco F	et al.	Exploring the ADAM12 Expression in Clear Cell Renal Cell Carcinoma: A Radiogenomic Analysis on CT Imaging	2024	Articles missing one or more keywords
Grenda R	et al.	Urinary excretion of endothelin-1 (ET-1), transforming growth factor-β1 (TGF-β1) and vascular endothelial growth factor (VEGF 165) in paediatric chronic kidney diseases: Results of the ESCAPE trial	2007	Articles missing one or more keywords
Grlić S	et al.	Single-Center Experience of Pediatric Cystic Kidney Disease and Literature Review	2024	Articles missing one or more keywords
Groen In 't Woud S	et al.	Compensatory Hypertrophy in Paediatric Patients with a Unilateral Ureteropelvic Junction Obstruction	2021	Articles missing one or more keywords
Guo YN	et al.	Colour Doppler and Biomarkers Utility for Renal Damage due to Congenital Hydronephrosis	2019	No Open Access
Gupta S	et al.	Trans IL-6 signaling does not appear to play a role in renal scarring after urinary tract infection	2020	Articles missing one or more keywords

Gupta S	et al.	Urinary antimicrobial peptides: Potential novel biomarkers of obstructive uropathy	2018	Articles missing one or more keywords
Gupta V	et al.	Correlation of Ablation Volume with Renal Function Loss after Cryoablation in Solitary Functioning Kidneys	2024	Articles missing one or more keywords
Gurioli A	et al.	Two cases of retroperitoneal metastasis from a completely regressed burned-out testicular cancer	2013	Articles missing one or more keywords
Gwak C	et al.	Preoperative C-reactive protein to albumin ratio as a novel prognostic biomarker for the oncological outcomes of radical nephroureterectomy	2024	Articles missing one or more keywords
Hamad J	et al.	Bladder preservation in muscle-invasive bladder cancer: a comprehensive review	2020	Review
Harris M	et al.	Genetic testing and biomarkers as predictive tools for congenital anomalies of kidney and urinary tract (Cakut)	2021	Conference Abstract
Hasslacher J	et al.	Insufficient performance of serum cystatin C as a biomarker for acute kidney injury of postrenal etiology	2012	Letter
Hassoun EA	et al.	Modulation of TCDD-induced fetotoxicity and oxidative stress in embryonic and placental tissues of C57BL/6J mice by vitamin E succinate and ellagic acid	1997	Articles missing one or more keywords
He Z	et al.	[Urine metabolomics analysis based on ultra performance liquid chromatography-high resolution mass spectrometry combined with osmolality calibration sample concentration variability]	2021	Articles missing one or more keywords
Heer R	et al.	Photodynamic versus white-light-guided resection of first-diagnosis non-muscleinvasive bladder cancer: PHOTO RCT	2022	Articles missing one or more keywords
Henriksson J	et al.	A prospective randomized trial on the effect of using an electronic monitoring drug dispensing device to improve adherence and compliance	2016	Articles missing one or more keywords
Hermanns T	et al.	Pre-treatment neutrophil-to-lymphocyte ratio as predictor of adverse outcomes in patients undergoing radical cystectomy for urothelial carcinoma of the bladder	2014	Articles missing one or more keywords
Hinojosa-Gonzalez D	et al.	Biomarkers in Urolithiasis	2023	Review
Hirselj DA	et al.	The megabladder mouse: A transgenic model for the study of obstructive uropathy	2009	Conference Abstract
Hosohata K	et al.	Vanin-1 in renal pelvic urine reflects kidney injury in a rat model of hydronephrosis	2018	No Human
How G	et al.	Neuronal defects an etiological factor in congenital pelviureteric junction obstruction?	2018	No Open Access
Hu C	et al.	Rare coexistence of mediastinal hepatoid adenocarcinoma, idiopathic azoospermia and horseshoe kidney: A case report and review of the literature	2015	Articles missing one or more keywords
Huang Q	et al.	Predictors of intestinal pseudo-obstruction in systemic lupus erythematosus complicated by digestive manifestations: Data from a Southern China lupus cohort	2016	Articles missing one or more keywords

Huang Y	et al.	Impact of AIB1 expression on the prognosis of upper tract urothelial carcinoma after radical nephroureterectomy	2019	Articles missing one or more keywords
Hubers L	et al.	How to Diagnose Immunoglobulin G4-Associated Cholangitis: The Jack-of-All-Trades in the Biliary Tract	2015	Review
Huda S	et al.	Immunoglobulin G-4-Related Retroperitoneal Fibrosis	2021	Articles missing one or more keywords
Ichino M	et al.	Increased Urinary Neutrophil Gelatinase Associated Lipocalin Levels in a Rat Model of Upper Urinary Tract Infection	2009	Articles missing one or more keywords
Ichino M	et al.	Urinary Neutrophil-Gelatinase Associated Lipocalin is a Potential Noninvasive Marker for Renal Scarring in Patients With Vesicoureteral Reflux	2010	No Open Access
Ichino M	et al.	Global gene expression profiling of renal scarring in a rat model of pyelonephritis	2008	Articles missing one or more keywords
Iida T	et al.	Small cell neuroendocrine carcinoma of the endometrium with difficulty identifying the original site in the uterus	2020	Articles missing one or more keywords
Im Y	et al.	Role of urine n-acetyl-glucosaminidase in predicting the prognosis of congential hydronephrosis	2020	Conference Abstract
Inoue S	et al.	Expression of phospho-elk1 and its prognostic significance in urothelial carcinoma of the upper urinary tract	2018	Articles missing one or more keywords
Irtan S	et al.	Wilms tumor: “State-of-the-art” update, 2016	2016	Articles missing one or more keywords
Isali I	et al.	A systematic review of underlying genetic factors associated with ureteropelvic junction obstruction in stenotic human tissue	2022	Review
Islam MI	et al.	SEPSIS â€“ HEMOPHAGOCYTIC LYMPHOHISTIOCYTOSIS OVERLAP: A RARE OVERLOOKED ENTITY AND ROLE OF PLASMAPHERESIS	2020	Conference Abstract
Itami Y	et al.	Preoperative predictive factors focused on inflammation-, nutrition-, and muscle-status in patients with upper urinary tract urothelial carcinoma undergoing nephroureterectomy	2019	Articles missing one or more keywords
Jackson AR	et al.	Roles for urothelium in normal and aberrant urinary tract development	2020	Articles missing one or more keywords
Jackson L	et al.	The molecular biology of pelvi-ureteric junction obstruction	2018	No Human
Jain S	et al.	Expression profiles of congenital renal dysplasia reveal new insights into renal development and disease	2007	No Open Access
Jan H	et al.	Combination of Platelet-Lymphocyte Ratio and Monocyte-Lymphocyte Ratio as a New Promising Prognostic Factor in Upper Tract Urothelial Carcinoma With Large Tumor Sizes > 3 cm	2020	Articles missing one or more keywords
Jan H	et al.	Combination of the Preoperative Systemic Immune-Inflammation Index and Monocyte-Lymphocyte Ratio as a Novel Prognostic Factor in Patients with Upper-Tract Urothelial Carcinoma	2019	Articles missing one or more keywords
Jankauskiene A	et al.	Editorial: CAKUT in Children and Adolescents: Towards Better Understanding of Impact and Risk Reduction	2022	Editorial

Ji X	et al.	Anti-sulfatide antibody-related Guillain–Barré syndrome presenting with overlapping syndromes or severe pyramidal tract damage: a case report and literature review	2024	Articles missing one or more keywords
Jiang H	et al.	A novel ETV6-NTRK3 gene fusion in primary renal fibrosarcoma	2022	Articles missing one or more keywords
Jianguo W	et al.	Serum and urinary procollagen III aminoterminal propeptide as a biomarker of obstructive nephropathy in children	2014	Articles missing one or more keywords
Jobs K	et al.	IL18 and NGAL in assessment of the risk of contrast induced nephropathy in children	2016	Conference Abstract
Joseph A	et al.	Hypoparathyroidism, Sensorineural deafness and renal disease (Barakat syndrome) caused by a reduced gene dosage in GATA3: A case report and review of literature	2019	Articles missing one or more keywords
Joseph D	et al.	Candidate urinary biomarker discovery in ureteropelvic junction obstruction	2010	No Open Access
Joseph N	et al.	Pre-treatment lymphocytopaenia is an adverse prognostic biomarker in muscle-invasive and advanced bladder cancer	2016	Articles missing one or more keywords
Joshi M	et al.	Concurrent durvalumab and radiation therapy (DUART) followed by adjuvant durvalumab in patients with localized urothelial cancer of bladder: Results from phase II study, BTCRC-GU15-023	2023	Articles missing one or more keywords
Kajbafzadeh AM	et al.	Urinary and Serum Carbohydrate Antigen 19-9 as a Biomarker in Ureteropelvic Junction Obstruction in Children	2010	No Open Access
Kantere D	et al.	Clinical features, complications and autoimmunity in male lichen sclerosus	2017	Articles missing one or more keywords
Kapoor K	et al.	Immunocytochemical detection of minichromosome maintenance protein 2 as a potential urinary-based marker of bladder cancer: A prospective observational study	2020	Articles missing one or more keywords
Kashiwagi Y	et al.	A case of chronic kidney disease with refractory periodic vomiting and hypertension in a pediatric patient	2025	Articles missing one or more keywords
Katims A	et al.	Feasibility and tissue concordance of genomic sequencing of urinary cytology in upper tract urothelial carcinoma	2023	Articles missing one or more keywords
Katsoufis CP	et al.	Clinical predictors of chronic kidney disease in congenital lower urinary tract obstruction	2020	Articles missing one or more keywords
Kawano M	et al.	IgG4-related kidney disease and retroperitoneal fibrosis: An update	2019	Review
Kawasaki T	et al.	A case of mucin-producing urothelial-type adenocarcinoma of the prostate showing immunoreactivity for NKX3.1, a specific marker of prostatic tissue	2017	Letter
Kelam N	et al.	Immunohistochemical Expression Pattern of FGFR1, FGFR2, RIP5, and HIP2 in Developing and Postnatal Kidneys of Dab1(-/-) (yotari) Mice	2022	Articles missing one or more keywords
Keller MB	et al.	Post-operative anuria due to a combination of urinoma and acute tubular necrosis after retroperitoneal tumor resection	2017	Conference Abstract
Kenfield S	et al.	PRESCRIPTION EXERCISE FOR OLDER MEN WITH URINARY DISEASE (PROUD) TRIAL	2024	Conference Abstract

Kerr K	et al.	Differential methylation as a diagnostic biomarker of rare renal diseases: A systematic review	2019	Review
Keshri R	et al.	Can urinary biomarkers be used in the outcome assessment of pyeloplasty in children?	2021	Articles missing one or more keywords
Khalil M	et al.	Key performance indicators for monitoring of anemia management and iron status in children attending a pediatric dialysis unit: the experience of Ain Shams University	2024	Articles missing one or more keywords
Khan M	et al.	Extensive ulcerated lesions in a patient with cutaneous myeloid sarcoma responded to twice-daily fludarabine and cytarabine regimen	2018	Letter
Khene Z	et al.	Risk stratification for kidney sparing procedure in upper tract urothelial carcinoma	2016	Review
Kihara M	et al.	Clinical significance of complement as a biomarker of disease activity in 4 cases of IgG4-related disease with retroperitoneal fibrosis	2013	Articles missing one or more keywords
Kim B	et al.	Plasma neutrophil gelatinase-associated lipocalin: a marker of acute pyelonephritis in children	2017	Articles missing one or more keywords
Kim B	et al.	Plasma Neutrophil Gelatinase-Associated Lipocalin	2015	Conference Abstract
Kim J	et al.	Ocular Involvement in the Histiocytoses: A Literature Review with an Illustrative Case Series	2022	Review
Kim J	et al.	Quest of biomarkers to predict the progression of chronic kidney diseases in children with CAKUT of Korea; a report from KNOW-Ped CKD	2023	Conference Abstract
Kim Y	et al.	Triple-Negative Lobular Breast Cancer Causing Hydronephrosis	2020	Articles missing one or more keywords
Kim YU	et al.	THE ROLE OF PRESEPSIN AS PREDICTIVE FACTOR FOR SEPSIS IN PATIENTS WITH URINARY TRACT INFECTION ASSOCIATED WITH URINARY CALCULI	2022	Conference Abstract
Kitsera N	et al.	Woman with turner syndrome and her child with acute leukemia (a case report)	2020	Articles missing one or more keywords
Kizilbash Q	et al.	Successful management of acute interstitial nephritis in two cases of disseminated tuberculosis	2016	Articles missing one or more keywords
Klatte T	et al.	Development of a preoperative nomogram incorporating biomarkers of systemic inflammatory response to predict non-organ-confined urothelial carcinoma of the bladder at radical cystectomy	2016	Conference Abstract
Klaus R	et al.	Biomarkers in ureteropelvic junction obstruction: Review and perspectives	2023	Review
Klein J	et al.	Clinical proteomics in obstetrics and neonatology	2014	Review
Klein J	et al.	Amniotic fluid peptide biomarkers for in utero prediction of postnatal renal function in cakut	2017	Conference Abstract
Klein J	et al.	The role of urinary peptidomics in kidney disease research	2016	Review

Klein J	et al.	Fetal urinary peptides to predict postnatal outcome of renal disease in fetuses with posterior urethral valves (PUV)	2013	Review
Klein J	et al.	Identification of prognostic markers of post-natal renal function in bilateral obstructive nephropathy by foetal urine proteome analysis	2012	Conference Abstract
Klemm J	et al.	Impact of Preoperative Plasma Potassium Levels on Oncological Outcomes, Major Complications, and 30-Day Mortality in Bladder Cancer Patients Undergoing Radical Cystectomy	2024	Articles missing one or more keywords
Ko Y	et al.	Procalcitonin determined at emergency department as an early indicator of progression to septic shock in patient with sepsis associated with ureteral calculi	2016	Articles missing one or more keywords
Kobayashi G	et al.	Cytological and histological findings of upper tract mucinous urothelial carcinoma with clear cell component: A case report and review of literature	2022	Articles missing one or more keywords
Kommoss F	et al.	Spindle Cell Sarcoma of the Uterine Corpus with Adipose Metaplasia: Expanding the Morphologic Spectrum of Neoplasms with MEIS1-NCOA2 Gene Fusion	2022	Articles missing one or more keywords
Kondo A	et al.	Segmental membranous nephropathy with severe IgG3 deposition	2018	Note
Konosu-Fukaya S	et al.	Renal epithelioid angiomyolipoma with malignant features: Histological evaluation and novel immunohistochemical findings	2014	Articles missing one or more keywords
Kool R	et al.	Role of Serum Lymphocyte-derived Biomarkers in Nonmetastatic Muscle-invasive Bladder Cancer Patients Treated with Trimodal Therapy	2021	Articles missing one or more keywords
Korecka K	et al.	Proteomics of urinary small extracellular vesicles in early diagnosis of kidney diseases in children-expectations and limitations	2024	Review
Kostadinova E	et al.	Potential role of cytokines in congenital malfomation of kidney and urinary tract	2018	Review
Kotha NV	et al.	Patterns of Failure After Definitive Chemoradiation for Muscle-Invasive Bladder Cancer	2021	Conference Abstract
Koukourikis P	et al.	Urine Biomarkers in the Management of Adult Neurogenic Lower Urinary Tract Dysfunction: A Systematic Review	2023	Review
Kramann R	et al.	Mouse models of kidney fibrosis	2021	Book chapter
Krishnan N	et al.	Molecular biomarkers in urine which may be useful as early markers of progressive kidney damage in congenital uropathy	2024	Conference Abstract
Krzemień G	et al.	Serum neutrophil gelatinase-associated lipocalin for predicting acute pyelonephritis in infants with urinary tract infection	2019	Articles missing one or more keywords
Kumari N	et al.	Primary Renal Well-Differentiated Neuroendocrine Tumors: Analyis of Six Cases from a Tertiary Care Center in North India with Review of Literature	2023	Articles missing one or more keywords
Kutluğ S	et al.	Vesicourethral reflux-induced renal failure in a patient with ICF syndrome due to a novel DNMT3B mutation	2016	No Open Access

Kuzovleva G	et al.	Urine metabolome investigation in pediatric urology. Review	2023	Review
Lacroix C	et al.	Label-free quantitative urinary proteomics identifies the arginase pathway as a new player in congenital obstructive nephropathy	2014	Articles missing one or more keywords
Lam JC	et al.	The utility of positron emission tomography (PET) for monitoring of management for IGG4 related kidney disease: A case study	2021	Conference Abstract
Lamoureux A	et al.	Symmetric dimethylarginirie for the early diagnosis of chronic kidney disease in a puppy	2017	Articles missing one or more keywords
Lee J	et al.	Associations of plasma neutrophil gelatinase-associated lipocalin, anemia, and renal scarring in children with febrile urinary tract infections	2020	Articles missing one or more keywords
Lee J	et al.	D-dimer as a marker of acute pyelonephritis in infants younger than 24 months with urinary tract infection	2018	Articles missing one or more keywords
Lee RS	et al.	Biomarkers for pediatric urological disease	2009	Review
Lenfant L	et al.	Current Evidence and Future Perspectives in the Management of Nonmetastatic Upper Tract Urothelial Carcinoma	2022	Articles missing one or more keywords
Leroy S	et al.	Procalcitonin, a useful biomarker in pediatric urinary tract infection	2013	Short survey
Levy A	et al.	Protein Expression Profiles among Lichen Sclerosus Urethral StricturesdCan Urethroplasty Success be Predicted?	2020	No Open Access
Levy A	et al.	Insights into the Pathophysiology of Urethral Stricture Disease due to Lichen Sclerosus: Comparison of Pathological Markers in Lichen Sclerosus Induced Strictures vs Nonlichen Sclerosus Induced Strictures	2019	No Open Access
Levy A	et al.	Pathophysiology of lichen sclerosus urethral strictures: The role of inflammation	2018	Conference Abstract
Li B	et al.	Screening for and Management of Chronic Kidney Disease for Children with Congenital Abnormalities of the Kidney and Urinary Tract	2018	Review
Li H	et al.	A novel signature to predict the neoadjuvant chemotherapy response of bladder carcinoma: Results from a territory multicenter real-world study	2022	Articles missing one or more keywords
Li H	et al.	Primary mucinous adenocarcinoma of the renal pelvis misdiagnosed as calculous pyonephrosis: A case report and literature review	2020	Review
Li Q	et al.	Clinical features and enzyme replacement therapy in 10 children with Fabry disease	2023	Articles missing one or more keywords
Li Q	et al.	What do we actually know about exosomal microRNAs in kidney diseases?	2022	Review
Li W	et al.	Acute kidney injury induced by various pneumoperitoneum pressures in a rabbit model of mild and severe hydronephrosis	2015	No human
Li X	et al.	Semaphorin-3A and Netrin-1 predict the development of kidney injury in children with congenital hydronephrosis	2018	No Open Access
Li Y	et al.	Disruption of Gen1 causes ectopic budding and kidney hypoplasia in mice	2022	No human

Li Y	et al.	Quantitative Proteome of Infant Stenotic Ureters Reveals Extracellular Matrix Organization and Oxidative Stress Dysregulation Underlying Ureteropelvic Junction Obstruction	2020	No Open Access
Li Z	et al.	Urinary heme oxygenase-1 in children with congenital hydronephrosis due to ureteropelvic junction obstruction	2012	No Open Access
Lichosik M	et al.	Interleukin 18 and neutrophil-gelatinase associated lipocalin in assessment of the risk of contrast-induced nephropathy in children	2015	Articles missing one or more keywords
Lin CY	et al.	Autotaxin early predicts progressive renal fibrosis in CKD	2019	Conference Abstract
Lin Y	et al.	Evaluation of pediatric hydronephrosis using deep learning quantification of fluid-to-kidney-area ratio by ultrasonography	2021	Articles missing one or more keywords
Liu J	et al.	Methylation status of RASSF1A gene promoter in upper tract urothelial carcinoma and its clinical significance	2016	Articles missing one or more keywords
Liu L	et al.	Editorial: Evidence and emerging option in diagnosis and management of upper tract urothelial carcinomas	2022	Editorial
Liu X	et al.	Peroxiredoxin i protein, a potential biomarker of hydronephrosis in fetal mice exposure to 2,3,7,8-tetrachlorodibenzo-p-dioxin	2014	No Human
Liu Y	et al.	Levofloxacin-induced crystal nephropathy	2015	Letter
Lopes R	et al.	Maternal Urinary Carbohydrate Antigen 19-9 as a Novel Biomarker for Evaluating Fetal Hydronephrosis: A Pilot Study (Urology 2016	2017	Letter
Lopes R	et al.	Serum and urinary values of CA 19-9 and TGFβ1 in a rat model of partial or complete ureteral obstruction	2015	No Human
Lughezzani G	et al.	Prognostic factors in upper urinary tract urothelial carcinomas: A comprehensive review of the current literature	2012	Review
Ma J	et al.	High RSK4 expression constitutes a predictor of poor prognosis for patients with clear cell renal carcinoma	2021	Articles missing one or more keywords
Macedo RV	et al.	WCN24-2115 Inflammatory Determinants and Associated Morbidity in Hemodialysis Patients	2024	Conference Abstract
Maciejczyk M	et al.	Salivary gland dysfunction, protein glycooxidation and nitrosative stress in children with chronic kidney disease	2020	Articles missing one or more keywords
Maciejczyk M	et al.	Salivary biomarkers of oxidative stress in children with chronic kidney disease	2018	Articles missing one or more keywords
Madsen M	et al.	Urinary biomarkers in hydronephrosis	2013	Review
Madsen MG	et al.	Urine and kidney cytokine profiles in experimental unilateral acute and chronic hydronephrosis	2012	No Open Access
Madsen MG	et al.	Urinary biomarkers in prenatally diagnosed unilateral hydronephrosis	2011	Review
Magalhães P	et al.	Urinary biomarkers for renal tract malformations	2016	Articles missing one or more keywords
Mahjoubi L	et al.	Personalized therapy based on sequential molecular analysis leads to 30 months of survival in a patient with diffuse unresectable gastric linitis plastica	2018	Articles missing one or more keywords

Majumder A	et al.	Renal angiomyoadenomatous tumor (RAT): a rare distinct entity with diagnostic challenges—a case report	2021	Articles missing one or more keywords
Makovetskaya GA	et al.	A child with newly diagnosed kidney disease and his family: determination of early factors of disease progression and features of nephroprotection	2023	Articles missing one or more keywords
Mamatov E	et al.	Predictive Role of Neutrophil Gelatinase-Associated Lipocaline in Donor-Specific Antibody-Positive and Donor-Specific Antibody-Negative Renal Transplant Patients	2015	Articles missing one or more keywords
Mann E	et al.	URINARY MICRORNAS ARE POTENTIAL BIOMARKERS OF SEVERITY OF URINARY TRACT DYSFUNCTION IN PATIENTS WITH POSTERIOR URETHRAL VALVES	2023	Conference Abstract
Mansour M	et al.	The diagnostic efficacy of diffusion tensor imaging in children with chronic kidney disease: correlation with histopathology and serum biomarkers	2024	Articles missing one or more keywords
Margel D	et al.	External validation of a biomarker based pre-cystectomy algorithm to predict nonorgan confined urothelial cancers	2012	Articles missing one or more keywords
Maritati F	et al.	Clinical and prognostic significance of serum IgG4 in chronic periaortitis. An analysis of 113 patients	2019	Articles missing one or more keywords
Marks SS	et al.	Renal blood flow measurements by magnetic resonance imaging using arterial spin labelling as a novel non-invasive biomarker in paediatric renal transplant recipients	2017	Conference Abstract
Martinez A	et al.	Extracephalic manifestations of nonchromosomal, nonsyndromic holoprosencephaly	2018	Review
Maryamchik E	et al.	Dedifferentiated Liposarcoma With Rhabdomyosarcomatous Differentiation Producing HCG: A Case Report of a Diagnostic Pitfall	2018	Articles missing one or more keywords
Mateo J	et al.	Olaparib in patients with metastatic castration-resistant prostate cancer with DNA repair gene aberrations (TOPARP-B): a multicentre, open-label, randomised, phase 2 trial	2020	Articles missing one or more keywords
Matsell DG	et al.	Predicting outcomes and improving care in children with congenital kidney anomalies	2020	Note
Matsell DG	et al.	Congenital Urinary Tract Obstruction-Diagnosis and Management in the Fetus	2018	Book chapter
Matsell DG	et al.	Developmental kidney injury predicts long-term outcome in boys with posterior urethral valves	2013	Conference Abstract
Matsuki M	et al.	The discrepancy between serum creatinine and cystatin C can predict renal function after treatment for postrenal acute kidney injury: multicenter study and pooled data analysis	2017	Articles missing one or more keywords
Matsumoto M	et al.	Renal insufficiency mimicking glutaric acidemia type 1 on newborn screening	2018	Articles missing one or more keywords
Matsuoka H	et al.	The long-term prognosis of nephropathy in operated reflux	2019	Articles missing one or more keywords
Maurya V	et al.	Tethered Cord Syndrome–A Study of the Short-Term Effects of Surgical Detethering on	2016	Articles missing one or more keywords

		Markers of Neuronal Injury and Electrophysiologic Parameters		
Mbeutcha A	et al.	Predictive models and prognostic factors for upper tract urothelial carcinoma: A comprehensive review of the literature	2016	Review
Mbeutcha A	et al.	Prognostic factors and predictive tools for upper tract urothelial carcinoma: a systematic review	2017	Review
Meeks J	et al.	Penile cancer biomarkers in men with lichen sclerosus	2011	Conference Abstract
Mello MF	et al.	Urinary extracellular matrix proteins as predictors of the severity of ureteropelvic junction obstruction in children	2021	No Open Access
Meng X	et al.	OSCA-finder: Redefining the assay of kidney disease diagnostic through metabolomics and deep learning	2023	Articles missing one or more keywords
Merchant S	et al.	Tuberculosis of the genitourinary system-Urinary tract tuberculosis: Renal tuberculosis-Part II	2013	Articles missing one or more keywords
Merz L	et al.	Urinary DKK3 - a potential long-term biomarker for progressive chronic kidney disease in children	2024	Conference Abstract
Mesrobian HGO	et al.	Urinary proteome analysis in patients with stable SFU grade 4 ureteropelvic junction obstruction differs from normal	2013	Note
Mesrobian HGO	et al.	Candidate urinary biomarker discovery in ureteropelvic junction obstruction: A proteomic approach	2010	No Open Access
Mesrobian HGO	et al.	The value of newborn urinary proteome analysis in the evaluation and management of ureteropelvic junction obstruction: a cost-effectiveness study	2009	Irrelevant
Mesrobian HGO	et al.	Analysis of the whole urinary proteome of stable infants with grade 4 ureteropelvic junction obstruction: A forgotten majority	2011	Conference Abstract
Mesrobian HGO	et al.	Liquid chromatography/mass spectrometry urinary proteome analysis discriminates between healthy infants and age matched patients with unilateral grade 4 ureteropelvic junction obstruction	2009	Conference Abstract
Metzger J	et al.	Adapting mass spectrometry-based platforms for clinical proteomics applications: The capillary electrophoresis coupled mass spectrometry paradigm	2009	Review
Meza J	et al.	Methods for Evaluating Renal Function in Patients with Neurogenic Bladder	2023	Review
Millner R	et al.	Albuminuria in Pediatric Neurogenic Bladder: Identifying an Earlier Marker of Renal Disease	2019	Articles missing one or more keywords
Millner R	et al.	Urothelial injury markers are elevated in neurogenic bladder patients and correlate with the presence of hydronephrosis	2017	Conference Abstract
Minato H	et al.	Ureteral Metastasis of Colonic Adenocarcinoma with Enteroblastic Differentiation: A Rare Case to be Distinguished from Clear Cell Adenocarcinoma of the Urinary Tract	2023	Articles missing one or more keywords

Mintz I	et al.	Positive predictive value of CT urography for upper tract urothelial carcinoma diagnosis using diagnostic ureteroscopy as the reference standard	2017	Conference Abstract
Miranda EP	et al.	The role of urinary KIM-1, NGAL, CA19-9 and β2-microglobulin in the assessment of ureteropelvic junction obstruction in adults	2017	No Open Access
Miranda EP	et al.	Diagnostic accuracy of urinary B2-microglobulin	2016	Conference Abstract
Mischak H	et al.	Urinary Proteomics Based on Capillary Electrophoresis-Coupled Mass Spectrometry in Kidney Disease: Discovery and Validation of Biomarkers, and Clinical Application	2010	Review
Mishra S	et al.	Lipoleiomyoma of the left broad ligament with dermoid cyst in ipsilateral ovary and synchronous multiple benign lesions of female genital tract: An unusual association	2016	Articles missing one or more keywords
Miteva L	et al.	Serum level of IL-10 and IL-12P40 in children with congenital malformation of kidney and urinary tract	2018	Articles missing one or more keywords
Miyamoto T	et al.	Assessment of type I interferon signatures in undifferentiated inflammatory diseases: A Japanese multicenter experience	2022	Articles missing one or more keywords
Miyata Y	et al.	A review of oxidative stress and urinary dysfunction caused by bladder outlet obstruction and treatments using antioxidants	2019	Review
Mohkam M	et al.	Novel urinary biomarkers for diagnosis of acute pyelonephritis in children	2020	Articles missing one or more keywords
Mongia A	et al.	Urine Biochemistry of a Human Fetus with Urinary Tract Obstruction–A Case Report	2023	No Open Access
Mooi J	et al.	Dual antiangiogenesis agents bevacizumab plus trebananib, without chemotherapy, in first-line treatment of metastatic colorectal cancer: Results of a phase II study	2021	Articles missing one or more keywords
Morizane S	et al.	Preoperative prognostic factors after radical nephroureterectomy in patients with upper urinary tract urothelial carcinoma	2013	Articles missing one or more keywords
Morones G	et al.	Vesicoureteral reflux: From diagnosis to treatment	2024	Book chapter
Morozov D	et al.	Urinary indicators of inflammation and fibrosis in children with congenital uropathies	2018	Conference Abstract
Morozova OL	et al.	Reflux nephropathy in children: Pathogenesis and prognosis	2021	Review
Morozova OL	et al.	Mechanisms of nephrosclerosis development in children with vesicoureteral reflux	2018	Review
Morozova OL	et al.	TGF-β1 and VEGF as biomarkers for renal scarring in children with vesico-ureteral reflux	2019	Conference Abstract
Morozova OL	et al.	Reflux nephropathy in children: early diagnosis and monitoring	2017	Review
Mortazavi F	et al.	Usefulness of serum Procalcitonin level for prediction of vesicoureteral reflux in pediatric urinary tract infection	2014	No Open Access
Motala F	et al.	Concurrent chemo-radiation induced renal and haematological toxicities in patients with invasive cervical cancer undergoing treatment	2022	Articles missing one or more keywords

Mueller T	et al.	Plasma concentrations of novel cardiac biomarkers before and after hemodialysis session	2015	Articles missing one or more keywords
Mukherjee E	et al.	Endothelial marker-expressing stromal cells are critical for kidney formation	2017	Articles missing one or more keywords
MurgiÄ‡ J	et al.	Contemporary bladder-sparing management of muscle-invasive bladder cancer-oncologist standpoint	2021	Conference Abstract
Murthi BS	et al.	18F-FDG PET/CT findings of Erdheim Chester disease a rare inflammatory condition - a pictorial presentation	2023	Conference Abstract
Musangile F	et al.	Targeted Next-Generation Sequencing of Flat Urothelial Lesions Reveals Putative Pathobiological Pathways, Potential Biomarkers, and Rational Therapeutic Targets	2023	Articles missing one or more keywords
Nabavizadeh B	et al.	Value of urinary carbohydrate antigen 19–9 to predict failure of conservative management in children with ureteropelvic junction obstruction	2019	No Open Access
Nadkarni M	et al.	Laboratory Findings After Urinary Tract Infection and Antimicrobial Prophylaxis in Children With Vesicoureteral Reflux	2020	No Open Access
Naik M	et al.	Epididymis-like Tubules in Adult Renal Hypodysplasia: Immunohistochemical Features Indicate a Mesonephric Origin	2017	No Open Access
Natalia Z	et al.	URINE BIOMARKERS AND REFLUX NEPHROPATHY (RN) IN CHILDREN WITH VESICOURETERAL REFLUX (VUR)	2022	Conference Abstract
Necchi A	et al.	A feasibility study of preoperative pembrolizumab before radical nephroureterectomy in patients with high-risk, upper tract urothelial carcinoma: PURE-02	2022	Articles missing one or more keywords
Nickavar A	et al.	Urine Neutrophil Gelatinase Associated Lipocalin as a Predictor of Vesicoureteral Reflux and Renal Parenchymal Damage: A Systematic Review	2023	Review
Nigam A	et al.	Impact of next generation sequencing on our understanding of CAKUT	2019	Review
Niles D	et al.	Evaluation of renal metabolic response to partial ureteral obstruction with hyperpolarized 13C MRI	2018	Articles missing one or more keywords
Nishiyama K	et al.	Resolution of Hydronephrosis in a Patient With Mucopolysaccharidosis Type II With Enzyme Replacement Therapy	2017	Articles missing one or more keywords
Novello R	et al.	MON-312 EVALUATION OF CHOLESTEROL AND TRIGLYCERIDES SERUM LEVELS IN PATIENTS ATTENDING TO A PEDIATRIC NEPHROLOGY AMBULATORY AT A SANTOS REFERENCE HOSPITAL	2019	Conference Abstract
Noyan A	et al.	Urinary NGAL, KIM-1 and L-FABP concentrations in antenatal hydronephrosis	2015	No Open Access
Ogbue O	et al.	Overview of histologic variants of urothelial carcinoma: current trends and narrative review on treatment outcomes	2022	Review
Oh K	et al.	The Role of Presepsin as Predictive Factor for Sepsis in Patients with Urinary Tract Infection Associated with Urinary Calculi	2022	Conference Abstract

Ohara Y	et al.	Phenotypic differences and similarities of monozygotic twins with maturity-onset diabetes of the young type 5	2019	Articles missing one or more keywords
Olvera-Posada D	et al.	KIM-1 Is a Potential Urinary Biomarker of Obstruction: Results from a Prospective Cohort Study	2017	No Open Access
Olvera-Posada D	et al.	KIM-1 is a potential urinary biomarker of obstruction: Results from a prospective cohort study in urological patients	2016	Conference Abstract
Olvera-Posada D	et al.	Kidney injury molecule-1 as a potential urinary biomarker of hydroneprosis may not be affected by inflammatory causes in the urinary tract	2017	Conference Abstract
Oshikawa-Hori S	et al.	Reduced urinary release of AQP1- and AQP2-bearing extracellular vesicles in patients with advanced chronic kidney disease	2021	Articles missing one or more keywords
Otero HJ	et al.	DTI of the kidney in children: comparison between normal kidneys and those with ureteropelvic junction (UPJ) obstruction	2020	Articles missing one or more keywords
Özgür S	et al.	Exploring the Predictive Role of Inflammatory Markers in Neuropathic Bladder-Related Kidney Damage with Machine Learning	2024	Articles missing one or more keywords
Ozkan S	et al.	Importance of neutrophil gelatinase-associated lipocalin in differential diagnosis of acute and chronic renal failure	2014	Articles missing one or more keywords
Özkuvancı i Ü	et al.	Response to Letter to the editor regarding "Can urinary biomarkers detect obstruction defined by renal functional loss in antenatal hydronephrosis?	2021	Letter
Özkuvancı Ü	et al.	Can urinary biomarkers detect obstruction defined by renal functional loss in antenatal hydronephrosis?	2020	No Open Access
Özsoy M	et al.	The Diagnostic Value of Soluble Triggering Receptor Expressed on Myeloid Cells for Patients with Acute Stone Pyelonephritis	2024	Articles missing one or more keywords
Pabuccu E	et al.	Does maternal hydronephrosis have an impact on urinary neutrophil gelatinase-associated lipocalin (uNGAL) levels?	2017	No Open Access
Palupi-Baroto R	et al.	Carotid intima-media thickness, fibroblast growth factor 23, and mineral bone disorder in children with chronic kidney disease	2024	Articles missing one or more keywords
Palupi-Baroto R	et al.	High fibroblast growth factor 23 as a biomarker for severe cardiac impairment in children with chronic kidney disease: A single tertiary center study	2021	Articles missing one or more keywords
Pan W	et al.	Research advancement on clinical evaluating modalities of renal scarring in children	2021	Review
Papachristou F	et al.	Urinary and serum biomarkers in ureteropelvic junction obstruction: a systematic review	2014	Review
Paraboschi I	et al.	Urinary biomarkers in pelvic-ureteric junction obstruction: a systematic review	2020	Review
Parad RB	et al.	Unreliability of standard fetal imaging biomarkers for prediction of lethal pulmonary hypoplasia (PH)	2019	Conference Abstract
Parikh C	et al.	Acute kidney injury: Defining prerenal azotemia in clinical practice and research	2010	Short survey

Parker C	et al.	Timing of radiotherapy after radical prostatectomy (RADICALS-RT): a randomised, controlled phase 3 trial	2020	Articles missing one or more keywords
Pather S	et al.	An expanded spectrum of high-grade B-cell non-hodgkin lymphomas involving the cervicovaginal region	2015	Articles missing one or more keywords
Pavlaki A	et al.	Letter to the editor regarding "Can urinary biomarkers detect obstruction defined by renal functional loss in antenatal hydronephrosis?	2021	Letter
Pavlaki A	et al.	Letter to the editor regarding "Can urinary biomarkers detect obstruction defined by renal functional loss in antenatal hydronephrosis?" by Ünsal Özkuvancı, M. İrfan Dönmez, Orhan Ziylan, Tayfun Oktar, Canan Küçükgergin, Bilal Çetin, Selçuk Erdem, Şule Seçkin	2021	Letter
Pavlaki A	et al.	Serum-Targeted HILIC-MS Metabolomics-Based Analysis in Infants with Ureteropelvic Junction Obstruction	2020	No Open Access
Pavlaki A	et al.	Matrix metalloproteinases in infants with ureteropelvic junction obstruction	2017	Conference Abstract
Pejchinovski M	et al.	Capillary zone electrophoresis on-line coupled to mass spectrometry: A perspective application for clinical proteomics	2015	Review
Pejcic M	et al.	Urinary proteomicsa tool for biomarker discovery	2010	Review
Peleli M	et al.	Renal denervation attenuates NADPH oxidase-mediated oxidative stress and hypertension in rats with hydronephrosis	2015	No Human
Pelizzo G	et al.	Discovering genotype variants in an infant with vacterl through clinical exome sequencing: A support for personalized risk assessment and disease prevention	2021	Articles missing one or more keywords
Penick E	et al.	Utilization of tumor markers in adnexal masses: A review of current literature	2019	Review
Perco P	et al.	Integrative analysis of -omics data and histologic scoring in renal disease and transplantation: Renal histogenomics	2010	Articles missing one or more keywords
Pérez-López L	et al.	Kidney function and glucose metabolism in overweight and obese cats	2020	No Human
Peyronnet B	et al.	Urinary TIMP-2 and MMP-2 are significantly associated with poor bladder compliance in adult patients with spina bifida	2019	Articles missing one or more keywords
Pezzicoli G	et al.	Concomitant Immunotherapy and Metastasis-Directed Radiotherapy in Upper Tract Urothelial Carcinoma: A Biomarker-Driven, Original, Case-Based Proof-of-Concept Study	2023	Articles missing one or more keywords
Piepsz A	et al.	Re: Biomarkers of congenital obstructive nephropathy: Past, present and future [5] (multiple letters)	2005	Erratum
Pierucci UM	et al.	Antenatal Determinants of Postnatal Renal Function in Fetal Megacystis: A Systematic Review	2024	Review
PinarbaÅŸi AS	et al.	URINE DKK3 AND CHRONIC KIDNEY DISEASE PROGRESSION IN CHILDREN WITH CAKUT	2022	Conference Abstract
Pinheiro AM	et al.	Neutrophil-to-lymphocyte ratio as biomarker for predicting locally advanced disease and	2020	Conference Abstract

		survival in patients treated with radical cystectomy		
Post A	et al.	Whole-body arginine dimethylation is associated with all-cause mortality in adult renal transplant recipients	2021	Articles missing one or more keywords
Prasad M	et al.	Imaging studies and biomarkers to detect clinically meaningful vesicoureteral reflux	2017	Review
Prestes TRR	et al.	The protective arm of the renin angiotensin system may counteract the intense inflammatory process in fetuses with posterior urethral valves	2016	Conference Abstract
Protti G	et al.	The methylome of buccal epithelial cells is influenced by age, sex, and physiological properties	2023	Articles missing one or more keywords
Quintavalla F	et al.	Blood plasma and urinary biomarkers of oxidative stress in cats with urethral obstruction	2024	No Human
Raaijmakers A	et al.	Clinical case of the year: A fetus with lower urinary tract obstruction	2015	Conference Abstract
Raina P	et al.	MN/CA9 gene expression as a potential tumor marker for renal cell carcinoma	2022	Articles missing one or more keywords
Ramirez-Gonzalez J	et al.	Assessment of the Relationship Between Inflammation and Glomerular Filtration Rate	2023	Articles missing one or more keywords
Rau S	et al.	Neutrophil gelatinase-associated lipocalin and end-stage renal disease: It is not all about the kidneys!	2013	Articles missing one or more keywords
Rdzanek O	et al.	Bilateral Wilms Tumor-Case Report of a Patient with Family History	2024	Articles missing one or more keywords
Rey-Cárdenas M	et al.	Digging into phenotype change in mismatch repair deficient endometrial carcinoma and treatment with immune checkpoint inhibition, a case report	2023	Articles missing one or more keywords
Ricci P	et al.	Urinary proteome signature of Renal Cysts and Diabetes syndrome in children	2019	Articles missing one or more keywords
Riccio S	et al.	New Insights from Metabolomics in Pediatric Renal Diseases	2022	Articles missing one or more keywords
Richards TL	et al.	Effects of transforming growth factor-beta (TGFÎ²) receptor i inhibition on renal biomarkers and fibrosis in unilateral ureteral occluded (UUO) Mice.	2017	Conference Abstract
Rodríguez-Suárez E	et al.	Urine as a source for clinical proteome analysis: From discovery to clinical application	2014	Review
Rosenblum S	et al.	Renal development in the fetus and premature infant	2017	Articles missing one or more keywords
Ruas A	et al.	Acute kidney injury in pediatrics: an overview focusing on pathophysiology	2022	Articles missing one or more keywords
Rudnik B	et al.	Upper Tract Urothelial Carcinoma	2021	Book chapter
Salamon RJ	et al.	Characterization of human iPSC RET reporter cell line differentiation to kidney and neural crest lineages	2018	Conference Abstract
Saltzman GB	et al.	Investigation of a non-invasive diagnostic assay to detect altered expression of microRNA in exfoliated urothelial carcinoma cells	2015	Conference Abstract
Sampedro F	et al.	Computing quantitative indicators of structural renal damage in pediatric DMSA scans	2016	Articles missing one or more keywords

Sangeetha G	et al.	A cost-effectiveness model comparing urinary biomarkers with diuretic renogram in diagnosing ureteropelvic junction obstruction in children	2023	Review
Sangeetha G	et al.	Comparing accuracy of urinary biomarkers in differentiation of ureteropelvic junction obstruction from nonobstructive dilatation in children	2022	Review
Santos J	et al.	Comparison of findings using ultrasonography and cystoscopy in urogenital schistosomiasis in a public health centre in rural angola	2015	Articles missing one or more keywords
Sarin YK	et al.	Is it Always Necessary to Treat an Asymptomatic Hydronephrosis Due to Ureteropelvic Junction Obstruction?	2017	Review
Sasmaz M	et al.	The relationship between the severity of pain and stone size, hydronephrosis and laboratory parameters in renal colic attack	2019	Articles missing one or more keywords
Saunders J	et al.	Novel Exonic Deletions in TTC7A in a Newborn with Multiple Intestinal Atresia and Combined Immunodeficiency	2019	Letter
Sav N	et al.	Evaluation of cardiovascular risk in children with solitary functioning kidney	2022	Articles missing one or more keywords
Schaefer FS	et al.	Cardiovascular Phenotypes in Children with CKD: The 4C Study	2016	Articles missing one or more keywords
Schaefer FS	et al.	Safety and usage of darbepoetin alfa in children with chronic kidney disease: prospective registry study	2016	Articles missing one or more keywords
Schaefer FS	et al.	Association of serum soluble urokinase receptor levels with progression of kidney disease in children	2017	Articles missing one or more keywords
Schaefer FS	et al.	Soluble urokinase plasminogen activator receptor (SUPAR) serum levels predict progression of kidney disease in children	2017	Conference Abstract
Schaefer FS	et al.	Supar serum levels predict progression of kidney disease in children	2017	Conference Abstract
Schanstra JP	et al.	Fetal biomarkers for lower urinary tract obstruction secondary to posterior urethral valves	2024	Review
Schlueter A	et al.	Feline mesothelioma: case report and review of cytologic, immunocytochemical, histopathologic, and immunohistochemical findings	2021	Articles missing one or more keywords
Scholz B	et al.	Seromucinous borderline tumor of the testis—A case report	2017	Articles missing one or more keywords
Schutter R	et al.	MRI for diagnosis of post-renal transplant complications: current state-of-the-art and future perspectives	2020	Review
Scotland K	et al.	Identification of a potential biomarker for stent-induced ureteral dysfunction	2020	Conference Abstract
Seah J	et al.	Neutrophil-Lymphocyte Ratio and Pathological Response to Neoadjuvant Chemotherapy in Patients with Muscle-Invasive Bladder Cancer	2015	Articles missing one or more keywords
Segev G	et al.	Urinary cystatin B differentiates progressive versus stable IRIS Stage 1 chronic kidney disease in dogs	2023	Articles missing one or more keywords

Seifriedova Z	et al.	The use of biomarkers in the diagnosis and treatment of obstruction of the upper urinary tract in children	2022	Review
Semko S	et al.	A case of synchronous bilateral calyx urothelial carcinoma: Combined treatment approach	2019	Articles missing one or more keywords
Seven A	et al.	Is there a correlation between maternal serum TGF-β 1 levels and fetal hydronephrosis?	2016	No Open Access
Shafiee M	et al.	Post-transplantation presentation of ANCA-associated vasculitis: Granulomatosis with polyangitis	2018	Articles missing one or more keywords
Shahrokhi P	et al.	The utility of radiolabeled PSMA ligands for tumor imaging	2022	Review
Shahzad A	et al.	Interleukin 8 (IL-8)-a universal biomarker?	2010	Review
Shaikh N	et al.	Association of Renal Scarring with Number of Febrile Urinary Tract Infections in Children	2019	Articles missing one or more keywords
Shaikh N	et al.	Identifying children with vesicoureteral reflux: A comparison of 2 approaches	2012	No Open Access
Shapiro L	et al.	Reply to editorial comment on urinary biomarkers related to UPJO	2020	Letter
Shehata MA	et al.	Clinical value of C-reactive protein and erythrocyte sedimentation rate in advanced bladder cancer	2019	Conference Abstract
Shen Q	et al.	Progress in diagnosis and treatment of congenital anomalies of the kidney and urinary tract	2020	Review
Shida MEF	et al.	Could CA 19-9 be a useful biomarker in the diagnosis, prognosis, and prediction of adequate relief in lower urinary tract obstructions?	2022	No Human
Shokeir A	et al.	Role of Urinary Tubular Enzymes in Evaluation of Children With Ureteropelvic Junction Narrowing Under Conservative Management	2009	No Open Access
Si S	et al.	Genetically Determined Chronic Low-Grade Inflammation and Hundreds of Health Outcomes in the UK Biobank and the FinnGen Population: A Phenome-Wide Mendelian Randomization Study	2021	Articles missing one or more keywords
Sibarani J	et al.	Urinary cytochrome c and caspase-3 as novel biomarker of renal function impairment in unilateral ureteropelvic junction obstruction model of wistar rats	2020	No Human
Sigumonrong YH	et al.	Degree of kidney injury due to artificial pelvic-ureteric junction obstruction with level of neutrophil gelatinase-associated lipocalin, interleukin 18, and histopathological descriptions in Wistar: experimental research	2023	No Human
Silva AJD	et al.	Pediatric chronic kidney disease: blood cell count indexes as inflammation markers	2023	Articles missing one or more keywords
Simões e Silva AC	et al.	Chemokines as potential markers in pediatric renal diseases	2014	Review
Simões e Silva AC	et al.	Interactions between cytokines, congenital anomalies of kidney and urinary tract and chronic kidney disease	2013	Review
Sivanathan J	et al.	Book: Genetics for obstetricians and gynaecologists: Chapter: Genetic markers on ultrasound scan	2017	Review

Smith-Harrison LI	et al.	Current applications of in utero intervention for lower urinary tract obstruction	2015	Review
Snipaitiene A	et al.	Unusual case of chronic recurrent multifocal osteomyelitis	2018	Articles missing one or more keywords
Soeda S	et al.	Successful management of platinum-resistant ovarian cancer by weekly Nedaplatin followed by Olaparib: Three case reports	2020	Articles missing one or more keywords
Solarin A	et al.	Predictive Value of Initial Diagnostic Imaging for Congenital Ureteropelvic Junction Obstruction Requiring Pyeloplasty	2024	Conference Abstract
Soliman AA	et al.	New biomarkers in screening and diagnosis of vesicoureteral reflux	2012	Conference Abstract
Sone K	et al.	Recurrent malignant melanoma of the uterine cervix treated with anti-PD-1 antibodies and anti-CTLA-4 antibodies: A case report	2022	Articles missing one or more keywords
Soria F	et al.	Validation of pre-treatment risk stratification parameters according to eau guidelines on upper tract urothelial carcinoma (UTUC)	2018	Conference Abstract
Sorić Hosman I	et al.	A Systematic Review of the (Un)known Host Immune Response Biomarkers for Predicting Recurrence of Urinary Tract Infection	2022	Review
Sorić Hosman I	et al.	Predicting autosomal dominant polycystic kidney disease progression: review of promising Serum and urine biomarkers	2023	Review
Sorić Hosman I	et al.	Cathelicidin in Urinary Tract Diseases: Diagnostic, Prognostic and Therapeutic Potential of an Evolutionary Conserved Antimicrobial Protein	2024	Review
Sretenovic M	et al.	Prognostic value of preoperative De Ritis ratio on oncological outcomes in patients with muscle-invasive bladder cancer	2024	Articles missing one or more keywords
Srivastava T	et al.	Urinary prostaglandin E2 is a biomarker of early adaptive hyperfiltration in solitary functioning kidney	2020	Articles missing one or more keywords
Stalmach A	et al.	Recent advances in capillary electrophoresis coupled to mass spectrometry for clinical proteomic applications	2013	Articles missing one or more keywords
Stamou M	et al.	Unilateral renal agenesis as an early marker for genetic screening in Kallmann syndrome	2019	Letter
Stankovic A	et al.	Promising biomarkers in pediatric chronic kidney disease through the kaleidoscope of CAKUT background complexity	2021	Note
Stanworth M	et al.	Elucidating the roles of SOD3 correlated genes and reactive oxygen species in rare human diseases using a bioinformatic-ontology approach	2024	Articles missing one or more keywords
Stepczynska A	et al.	Implementation of CE-MS-identified proteome-based biomarker panels in drug development and patient management	2016	Review
Stodkilde L	et al.	Urinary proteome analysis in congenital bilateral hydronephrosis	2013	No Open Access
Sukumar J	et al.	Activating BRAF mutation in sclerosing mucoepidermoid carcinoma with eosinophilia of the thyroid gland: Two case reports and review of the literature	2019	Review
Sun J	et al.	Recent advances of ureteropelvic junction obstruction biomakers in children	2021	Review
Sung B	et al.	Chronic kidney disease in neurogenic bladder	2018	Articles missing one or more keywords

Suresh J	et al.	Evaluation of dynamic thiol-disulphide homeostasis in obstructive uropathy	2020	No Human
Tain YL	et al.	Cardiovascular Risks of Hypertension: Lessons from Children with Chronic Kidney Disease	2022	Review
Taj-Aldeen S	et al.	Serum cytokine profile in patients with candidemia versus bacteremia	2021	Articles missing one or more keywords
Takahashi H	et al.	Clinical Practice and Examination for IgG4-Related Disease	2015	Review
Tam F	et al.	Renal monocyte chemoattractant protein-1: An emerging universal biomarker and therapeutic target for kidney diseases?	2020	Review
Tamura R	et al.	Refractory ovarian squamous cell carcinoma arising from a seromucinous borderline tumor with squamous overgrowth: A case report	2024	Articles missing one or more keywords
Tanaka H	et al.	Bladder preservation therapy in muscle-invasive bladder cancer: Current evidence and future perspectives	2020	Review
Tanaka MAB	et al.	Urinary markers in children and adolescents with vesicoureteral reflux	2016	Conference Abstract
Tandogdu Z	et al.	Management of the Urologic Sepsis Syndrome	2016	Review
Tang K	et al.	Predictive value of preoperative inflammatory response biomarkers for metabolic syndrome and post-PCNL systemic inflammatory response syndrome in patients with nephrolithiasis	2017	Articles missing one or more keywords
Tao SY	et al.	THE USE OF URINARY BIOMARKERS IN PEDIATRIC PATIENTS WITH UNILATERAL HYDRONEPHROSIS TO IDENTIFY TRUE URETEROPELVIC JUNCTION OBSTRUCTION	2023	Conference Abstract
Taranta-Janusz K	et al.	Is urine intercellular adhesion molecule-1 a marker of renal disorder in children with ureteropelvic junction obstruction?	2016	No Open Access
Taranta-Janusz K	et al.	Osteopontin and symmetric dimethylarginine plasma levels in solitary functioning kidney in children	2012	Articles missing one or more keywords
Taranta-Janusz K	et al.	Urine exoglycosidases are potential markers of renal tubular injury in children with ureteropelvic junction obstruction	2015	No Open Access
Taranta-Janusz K	et al.	New tubular injury markers in children with solitary functioning kidney	2014	Conference Abstract
Taranta-Janusz K	et al.	Angiotensinogen as a novel marker of obstructive nephropathy in children.	2014	Conference Abstract
Taranta-Janusz K	et al.	KIM-1 and NGAL: New markers of obstructive nephropathy	2011	Conference Abstract
Taşdemir M	et al.	Urinary biomarkers in the early detection and follow-up of tubular injury in childhood urolithiasis	2018	Irrelevant
Terciu M	et al.	Early Outcome of Multisystem Inflammatory Syndrome in Neonates Diagnosed following Prenatal Maternal COVID-19 Infection: A Three-Case Series	2023	Articles missing one or more keywords
Theilen T	et al.	Multidisciplinary Treatment Strategies for Wilms Tumor: Recent Advances, Technical Innovations and Future Directions	2022	Review
Thorner P	et al.	Estrogen Receptor Expression in DICER1-related Lesions is Associated with the Presence of Cystic Components	2024	Articles missing one or more keywords

Thoulouzan M	et al.	Outcomes of GreenLight XPS-180 W laser photovaporization for BPH larger than 80 mL	2017	Articles missing one or more keywords
Tian F	et al.	Urinary Emmprin, matrix metalloproteinase 9 and tissue inhibitor of metalloproteinase 1 as potential biomarkers in children with ureteropelvic junction narrowing on conservative treatment	2015	No Open Access
Tiglao Jr E	et al.	Chronic obstructive pulmonary disease and pulmonary hypertension in a patient with klippel-feil syndrome (KFS): A rare association	2015	Conference Abstract
Tokarchuk N	et al.	FIBROTIC MARKERS IN INFANTS WITH PYELONEPHRITIS	2019	No English
Toker A	et al.	Is urinary kidney injury molecule-1 a noninvasive marker for renal scarring in children with vesicoureteral reflux?	2013	No Open Access
Tokuc E	et al.	Evaluation of dynamic thiol-disulphide homeostasis in obstructive uropathy	2020	No Human
Tolun AA	et al.	Clinical Untargeted Metabolomics and its Utility in Undiagnosed Cases: The First Case in Australia	2021	Conference Abstract
Tsao S	et al.	Krukenberg tumor with concomitant ipsilateral hydronephrosis and spermatic cord metastasis in a man: A case report	2021	Articles missing one or more keywords
Turcan D	et al.	Nephrogenic adenoma of the urinary tract: A 6-year single center experience	2017	Articles missing one or more keywords
Turco M	et al.	P0168 Urosepsis in malignant ureteral obstruction or obstructing stones: Is the treatment the same?	2021	Conference Abstract
Turedi S	et al.	Differences in ischemia-modified albumin levels between end stage renal disease patients and the normal population	2010	Articles missing one or more keywords
Ugur Yilmaz M	et al.	Does thiol-disulphide balance in tissue and serum play a role in disease management in ureteropelvic junction stenosis?	2024	No Open Access
Urbschat A	et al.	Serum and urinary NGAL but not KIM-1 raises in human postrenal AKI	2014	Articles missing one or more keywords
Urushibara M	et al.	Differential treatment responses to immune checkpoint inhibitor (ICI) therapy in a case of multiple primary malignancies: the programmed death ligand-1 (PD-L1) negative ureteral and lung metastasis from a clear cell renal cell carcinoma appearing after robotic-assisted partial nephrectomy progressed after ICI therapy, while synchronous PD-L1-positive primary lung squamous cell carcinoma responded very well to ICI therapy: a case report	2023	Articles missing one or more keywords
Uwaezuoke S	et al.	Posterior urethral valve in children: Using novel biomarkers as an early predictive tool for the onset and progression of chronic kidney disease	2022	Review
Uwaezuoke S	et al.	Congenital ureteropelvic junction obstruction: Physiopathology, decoupling of tout court pelvic dilatation-obstruction semantic connection, biomarkers to predict renal damage evolution	2012	Review
Vadasz Z	et al.	Overexpression of semaphorin 3A in patients with urothelial cancer	2018	Articles missing one or more keywords

Vaezzadeh A	et al.	Identification of novel urinary biomarkers of renal obstruction using temporal quantitative proteomics	2009	Conference Abstract
Valério FC	et al.	Biomarkers in vesicoureteral reflux: An overview	2020	Review
Van Der Ven A	et al.	A homozygous missense variant in VWA2, encoding an interactor of the Fraser-complex, in a patient with vesicoureteral reflux	2018	Articles missing one or more keywords
Van Roij K	et al.	Discrepant results of serum creatinine and cystatin C in a urological patient	2017	Short survey
Velpurisiva P	et al.	Overview of Biomarkers of Rejection in Pediatric Renal Transplantation	2023	Book chapter
Vemulakonda VM	et al.	Ureteropelvic junction obstruction: Diagnosis and management	2021	Review
Vendrig LM	et al.	Translational strategies to uncover the etiology of congenital anomalies of the kidney and urinary tract	2024	Review
Verhoest G	et al.	Predictive factors of recurrence and survival of upper tract urothelial carcinomas	2011	Articles missing one or more keywords
Vidaeff A	et al.	Preeclampsia: The Need for a Biological Definition and Diagnosis	2021	Review
Vig A	et al.	Role of Emerging Urinary Biomarkers in Predicting Progressive Deterioration of Kidney Function in Congenital Anomalies of Kidney and Urinary Tract: Trefoil Family Factor 3, Alpha Soluble Klotho and Urinary Microalbuminuria	2024	No Open Access
Viteri B	et al.	Ultrasound-Based Renal Parenchymal Area and Kidney Function Decline in Infants With Congenital Anomalies of the Kidney and Urinary Tract	2021	Review
Vitko D	et al.	Urine from the patients with vesicoureteral reflux reveals changes in host and bacterial metabolism after urinary tract infection	2019	Conference Abstract
Wakabayashi EA	et al.	Congenital Solitary Functioning Kidney: A Review	2023	Review
Walker EYX	et al.	Congenital anomalies of the kidney and urinary tract: antenatal diagnosis, management and counselling of families	2024	Review
Walker K	et al.	Deletion of FGFR2 from tailbud-derived stroma leads to vesicoureteral reflux	2013	Conference Abstract
Wang R	et al.	Prenatal diagnosis of dicentric chromosome X mosaicism: a case report and review	2024	Articles missing one or more keywords
Wang X	et al.	Defining the Urine Proteome in Boys with Posterior Urethral Valves	2024	Conference Abstract
Wang X	et al.	Supar induces proteinuria in solitary functioning kidney models	2017	Conference Abstract
Wang Z	et al.	Metabolic Response in Rabbit Urine to Occurrence and Relief of Unilateral Ureteral Obstruction	2018	No Human
Wang Z	et al.	Evolution of the urinary proteome during human renal development and maturation	2015	No Open Access
Washino S	et al.	Roles played by biomarkers of kidney injury in patients with upper urinary tract obstruction	2020	Review

Weiss A	et al.	Expansion of the renal capsular stroma, ureteric bud branching defects and cryptorchidism in mice with Wilms tumor 1 gene deletion in the stromal compartment of the developing kidney	2020	No Human
Weiss A	et al.	Delayed onset of smooth muscle cell differentiation leads to hydroureter formation in mice with conditional loss of the zinc finger transcription factor gene Gata2 in the ureteric mesenchyme	2019	No Human
Wilhide M	et al.	Renal epithelial miR-205 expression correlates with disease severity in a mouse model of congenital obstructive nephropathy	2016	No Human
Wu B	et al.	A retrospective cohort study on the effects of Down's screening markers and maternal characteristics on pregnancy outcomes in preeclampsia	2022	Articles missing one or more keywords
Wu B	et al.	Identification of transcripts associated with renal damage due to ureteral obstruction as candidate urinary biomarkers	2018	No Human
Wu B	et al.	Gene expression changes induced by unilateral ureteral obstruction in mice	2012	No Human
Wu C	et al.	The significance of neutrophil-to-lymphocyte ratio and combined chemoradiotherapy in patients undergoing bladder preservation therapy for muscle-invasive bladder cancer	2020	Articles missing one or more keywords
Wu L	et al.	Kidney transplantation after liver transplantation	2016	Articles missing one or more keywords
Xia Q	et al.	N-acetylcysteine ameliorates contrast-induced kidney injury in rats with unilateral hydronephrosis	2018	No Human
Xiao M	et al.	Renal-on-Chip Microfluidic Platform with a Force-Sensitive Resistor (ROC-FS) for Molecular Pathogenesis Analysis of Hydronephrosis	2022	No Open Access
Xiong G	et al.	Aristolochic acid containing herbs induce gender-related oncological differences in upper tract urothelial carcinoma patients	2018	Conference Abstract
Xiong G	et al.	Prognostic and predictive value of epigenetic biomarkers in upper tract urothelial carcinoma	2015	Conference Abstract
Xu J	et al.	Osr1 interacts synergistically with Wt1 to regulate kidney organogenesis	2016	Articles missing one or more keywords
Xu Z	et al.	Serum and urinary thioredoxin concentrations are associated with severity of children hydronephrosis	2017	No Open Access
Yamaguchi K	et al.	Phase 1b study of andecaliximab (GS-5745	2019	Conference Abstract
Yamamura-Miyazaki N	et al.	Factors associated with 1-year changes in serum fibroblast growth factor 23 levels in pediatric patients with chronic kidney disease	2022	Articles missing one or more keywords
Yamanouchi S	et al.	Reduced urinary excretion of neutrophil gelatinase-associated lipocalin as a risk factor for recurrence of febrile urinary tract infection in children	2021	Articles missing one or more keywords
Yamanouchi S	et al.	Urinary ngal as a risk factor for recurrence of febrile urinary tract infection in children	2018	Conference Abstract

Yang H	et al.	Aristolochic acid and immunotherapy for urothelial carcinoma: Directions for unmet needs	2019	Review
Yang J	et al.	Could a first-trimester blood phosphatidylethanol concentration ≥4nM be useful to identify women with moderate-to-heavy prenatal alcohol exposure who are at high risk of adverse pregnancy outcomes?	2015	Articles missing one or more keywords
Yang W	et al.	Roles of MMP7 and MMP9 in the pathogenesis of congenital ureteropelvic junction obstruction	2022	Conference Abstract
Yasuda H	et al.	Renal Function and Hematology in Rats with Congenital Renal Hypoplasia	2016	No Human
Yasuoka S	et al.	A case of primary malignant lymphoma of the prostate gland presenting as right lower back pain and dysuria	2018	Articles missing one or more keywords
Yates DR	et al.	Distinct patterns and behaviour of urothelial carcinoma with respect to anatomical location: How molecular biomarkers can augment clinico-pathological predictors in upper urinary tract tumours	2013	Review
Yazılıtaş F	et al.	The relevance of practical laboratory markers in predicting high-grade vesicoureteral reflux and renal scarring	2023	No Open Access
Yiğit D	et al.	Can serum Neutrophil Gelatinase Associated Lipocalin and Kidney Injury Molecule−1 help in decision making for surgery in antenatally dedected hydronephrosis	2021	No Open Access
Yilmaz A	et al.	Matrix metalloproteinase 9 and tissue inhibitor of metalloproteinase 1 in vesicoureteral reflux	2012	Conference Abstract
Yoon S	et al.	Predictive factors for bacteremia in febrile infants with urinary tract infection	2020	Articles missing one or more keywords
Yoshikawa A	et al.	Safety and tolerability of andecaliximab as monotherapy and in combination with an anti-PD-1 antibody in Japanese patients with gastric or gastroesophageal junction adenocarcinoma: A phase 1b study	2022	Articles missing one or more keywords
Yousefichaijan P	et al.	The effect of vitamin A on clinical manifestations of recurrent pyelonephritis in children	2020	Irrelevant
Yu S	et al.	Clinical biomarkers of long-term outcome in congenital urinary tract obstruction	2013	Conference Abstract
Yu Z	et al.	Prognostic and clinicopathological significance of systemic immune-inflammation index in upper tract urothelial carcinoma: a meta-analysis of 3911 patients	2024	Review
Yücel ÖB	et al.	Urinary biomarkers can identify the need for pyeloplasty in presence of supranormal differential renal function in antenatally diagnosed unilateral hydronephrosis	2022	No Open Access
Zacharias F	et al.	The role of microRNAs identified in the amniotic fluid	2020	Review
Zakiyanov O	et al.	Matrix metalloproteinases in renal diseases: A critical appraisal	2019	Review
Zganjar A	et al.	Diagnosis, workup, and risk stratification of upper tract urothelial carcinoma	2023	Review

Zhang B	et al.	Preoperative plasma fibrinogen level represents an independent prognostic factor in a Chinese cohort of patients with upper tract urothelial carcinoma	2016	Articles missing one or more keywords
Zhang H	et al.	Primary mucinous tumors of the renal pelvis: Clinical, histopathological, and molecular analysis of three cases	2024	Articles missing one or more keywords
Zhang K	et al.	3D bioprinting of urethra with PCL/PLCL blend and dual autologous cells in fibrin hydrogel: An in vitro evaluation of biomimetic mechanical property and cell growth environment	2017	Articles missing one or more keywords
Zhang M	et al.	A group of sclerosing epithelioid fibrosarcomas with low-level amplified EWSR1-CREB3L1 fusion gene in children	2022	Articles missing one or more keywords
Zhang Q	et al.	Quantification of the renal sinus fat and exploration of its relationship with ectopic fat deposition in normal subjects using MRI fat fraction mapping	2023	Articles missing one or more keywords
Zhao B	et al.	Finding the best subgroup with differential treatment effect with multiple outcomes	2024	Articles missing one or more keywords
Zhao Y	et al.	Predictive Value of the Platelet-Lymphocyte Ratio for Intravesical Recurrence After Radical Nephroureterectomy: A Retrospective Study	2024	Articles missing one or more keywords
Zheng Q	et al.	Computer-aided diagnosis of congenital abnormalities of the kidney and urinary tract in children based on ultrasound imaging data by integrating texture image features and deep transfer learning image features	2019	Articles missing one or more keywords
Zheng Y	et al.	The significance of Pax2 expression in the ureter epithelium of children with vesicoureteric reflux	2015	No Open Access
Zhian H	et al.	Urine metabolomics analysis based on ultra performance liquid chromatography-high resolution mass spectrometry combined with osmolality calibration sample concentration variability	2021	Articles missing one or more keywords
Zhou JX	et al.	Non-coding rnas in hereditary kidney disorders	2021	Review
Zhou R	et al.	The Neutrophil Gelatinase-Associated Lipocalin (NGAL) as a Biomarker for Monitoring Renal Function in Patients with Solitary Kidney Stones: A Single-Center, Retrospective Analysis of 23 Cases	2022	Articles missing one or more keywords
Zhu H	et al.	The Impact of Minimally Invasive Surgery on Treating Patients with Early Cervical Adenocarcinoma	2022	Articles missing one or more keywords
Zhu Z	et al.	Preoperative predictors of early death risk in bladder cancer patients treated with robot-assisted radical cystectomy	2019	Articles missing one or more keywords
Zong R	et al.	Overexpressed Histocompatibility Minor 13 was Associated with Liver Hepatocellular Carcinoma Progression and Prognosis	2022	Articles missing one or more keywords
	et al.	Indwelling ureteral stents induce ureteral aperistalsis injury and fibrosis	2019	Conference Abstract
	et al.	43rd Spanish Congress of Pediatric Nephrology	2019	Conference Review

Supplmentary File S3. List of included studies.

Authors		Title	Year	Doi
Amiri R	et al.	Evaluation of urinary neutrophil gelatinase-associated lipocalin as a biomarker in pediatric vesicoureteral reflux assessment	2020	
Anand S	et al.	Urinary biomarkers as point-of-care tests for predicting progressive deterioration of kidney function in congenital anomalies of kidney and urinary tract: trefoil family factors (TFFs) as the emerging biomarkers	2021	10.1007/s00467-020-04841-8
Atar A	et al.	The roles of serum and urinary carbohydrate antigen 19-9 in the management of patients with antenatal hydronephrosis	2015	10.1016/j.jpurol.2014.12.012
Bajpai M	et al.	The role of Plasma Renin Activity in prenatally diagnosed non-obstructed hydronephrosis at risk for surgery—an observational study	2022	10.1007/s40620-021-01199-4
Banerjee I	et al.	Role of urinary and serum carbohydrate antigen 19-9 as a biomarker in diagnosis of adult giant hydronephrosis	2016	10.7860/JCDR/2016/21400.8453
Bastos FM	et al.	Tubular and glomerular biomarkers of renal tissue function in the urine of fetuses with posterior urethral valves	2022	10.1016/j.jpurol.2022.03.019
Begou O	et al.	Diminished systemic amino acids metabolome and lipid peroxidation in ureteropelvic junction obstruction (Upjo) infants requiring surgery	2021	10.3390/jcm10071467
Benzer M	et al.	Urinary L-FABP as a marker of vesicoureteral reflux in children: could it also have a protective effect on the kidney?	2017	10.1007/s11255-016-1389-6
Bieniaś B	et al.	Selected Metal Matrix Metalloproteinases and Tissue Inhibitors of Metalloproteinases as Potential Biomarkers for Tubulointerstitial Fibrosis in Children with Unilateral Hydronephrosis	2020	10.1155/2020/9520309
Bieniaś B	et al.	Potential novel biomarkers of obstructive nephropathy in children with hydronephrosis	2018	10.1155/2018/1015726
Branco BC	et al.	Urinary HSP70 can predict the indication of surgery in unilateral ureteropelvic junction obstruction	2022	10.1007/s00383-021-05059-x
Bu L	et al.	Association between NDUFS1 from urinary extracellular vesicles and decreased differential renal function in children with ureteropelvic junction obstruction	2024	10.1186/s12882-024-03592-0
Buffin-Meyer B	et al.	Combination of the fetal urinary metabolome and peptidome for the prediction of postnatal renal outcome in fetuses with PUV	2018	10.1016/j.jprot.2018.06.012
Cetin N	et al.	Urine hepcidin, netrin-1, neutrophil gelatinase-associated lipocalin and C-C motif chemokine ligand 2 levels in multicystic dysplastic kidney	2020	10.1590/2175-8239-JBN-2019-0022
Chen H	et al.	Quantitative Urinary Proteome Reveals Potential Biomarkers for Ureteropelvic Junction Obstruction	2019	10.1002/prca.201800101
Colceriu MC	et al.	The Utility of Noninvasive Urinary Biomarkers for the Evaluation of Vesicoureteral Reflux in Children	2023	10.3390/ijms242417579
Cost N	et al.	Urinary NGAL levels correlate with differential renal function in patients with ureteropelvic junction obstruction undergoing pyeloplasty	2013	10.1016/j.juro.2013.05.003
de Sépibus R	et al.	Urinary albumin excretion and chronic kidney disease in children with vesicoureteral reflux	2017	10.1016/j.jpurol.2017.04.004
Decramer S	et al.	Non-invasive markers of ureteropelvic junction obstruction	2007	10.1007/s00345-007-0201-8
Devarakonda CKV	et al.	A novel urinary biomarker protein panel to identify children with ureteropelvic junction obstruction – A pilot study	2020	10.1016/j.jpurol.2020.05.163
Dreux S	et al.	Urine biochemistry to predict long-term outcomes in fetuses with posterior urethral valves	2018	10.1002/pd.5359
Fendereski K	et al.	Comparing predictive values of carbohydrate antigen 19-9, neutrophil gelatinase–associated lipocalin, and kidney injury molecule-1 in 161 patients with ureteropelvic junction obstruction	2021	10.1007/s00467-020-04750-w

Filipovic B	et al.	Benign hydronephrosis and elevated of serum levels of carbohydrate antigen CA 19-9: A case report	2016	10.12659/AJCR.897900
Ganapathy S	et al.	Comparison of diagnostic accuracy of models combining the renal biomarkers in predicting renal scarring in pediatric population with vesicoureteral reflux (VUR)	2023	10.1007/s11845-023-03275-z
Gao J	et al.	Comprehensive proteomic characterization of urethral stricture disease in the Chinese population	2024	10.3389/fmolb.2024.1401970
García-Nieto V	et al.	Renal tubular markers as screening tools for severe vesicoureteral reflux	2019	10.1007/s00431-019-03324-9
Gawłowska-Marciniak A	et al.	Evaluation of TGF-β1, CCL5/RANTES and sFas/Apo-1 urine concentration in children with ureteropelvic junction obstruction	2013	10.5114/aoms.2013.36912
Geminiganesan S	et al.	Comparison of Urinary Biomarkers in Diagnosis of Ureteropelvic Junction Obstruction and Differentiation from Nonobstructive Dilatation	2024	10.4103/jiaps.jiaps_204_23
Geraud N	et al.	Evaluation of predictive performance of fetal urinary inflammatory markers of postnatal kidney function in fetuses with posterior urethral valves	2024	10.1007/s00467-024-06608-x
Gerber C	et al.	Proximal tubule proteins are significantly elevated in bladder urine of patients with ureteropelvic junction obstruction and may represent novel biomarkers: A pilot study	2016	10.1016/j.jpurol.2015.10.008
Gül M	et al.	The predictive value of platelet to lymphocyte and neutrophil to lymphocyte ratio in determining urethral stricture after transurethral resection of prostate	2017	10.5152/tud.2017.14478
Gupta S	et al.	Impact of successful pediatric ureteropelvic junction obstruction surgery on urinary HIP/PAP and BD-1 levels	2020	10.1016/j.jpurol.2020.03.006
Jovanovic I	et al.	Transcriptome-wide based identification of miRs in congenital anomalies of the kidney and urinary tract (CAKUT) in children: The significant upregulation of tissue miR-144 expression	2016	10.1186/s12967-016-0955-0
Kajbafzadeh AM	et al.	Prognostic significance of maternal urinary carbohydrate antigen 19-9 for antenatal diagnosis of posterior urethral valve associated with fetal hydronephrosis	2019	10.1007/s11255-019-02138-w
Kajbafzadeh AM	et al.	Maternal Urinary Carbohydrate Antigen 19-9 as a Novel Biomarker for Evaluating Fetal Hydronephrosis: A Pilot Study	2017	10.1016/j.urology.2016.10.038
Karakus S	et al.	Urinary IP-10, MCP-1, NGAL, Cystatin-C, and KIM-1 Levels in Prenatally Diagnosed Unilateral Hydronephrosis: The Search for an Ideal Biomarker	2016	10.1016/j.urology.2015.09.007
Katsoufis CP	et al.	Risk assessment of severe congenital anomalies of the kidney and urinary tract (CAKUT): A birth cohort	2019	10.3389/fped.2019.00182
Kazlauskas V	et al.	Expression of tissue fibrosis genes in congenitally obstructed pyeloureteral junction and biomarkers of renal damage	2024	10.5173/ceju.2023.218R
Kazlauskas V	et al.	Urine Biomarkers Combined With Ultrasound for the Diagnosis of Obstruction in Pediatric Hydronephrosis	2022	10.3389/fped.2021.762417
Kohli H	et al.	Differential expression of miRNAs involved in biological processes responsible for inflammation and immune response in lichen sclerosus urethral stricture disease	2021	10.1371/journal.pone.0261505
Kostic D	et al.	Biomarkers for Early Detection of Renal Injury in Fetuses With Congenital Urinary Tract Obstruction	2022	10.3389/fruro.2022.883903
Kostic D	et al.	First-year profile of biomarkers for early detection of renal injury in infants with congenital urinary tract obstruction	2019	10.1007/s00467-019-4195-4
Kostic D	et al.	The role of renal biomarkers to predict the need of surgery in congenital urinary tract obstruction in infants	2019	10.1016/j.jpurol.2019.03.009
Li J	et al.	Urinary exosomal vitronectin predicts vesicoureteral reflux in patients with neurogenic bladders and spinal cord injuries	2022	10.3892/etm.2021.10988
Lin Y	et al.	Evaluation of pediatric hydronephrosis using deep learning quantification of fluid-to-kidney-area ratio by ultrasonography	2021	10.1007/s00261-021-03201-w
Liu CM	et al.	Use of Urine N-Terminal Prohormone of Brain-Natriuretic Peptide (NT-proBNP) as a Non-Invasive Indicator for Renal Function Recovery after Surgical Relief of Hydronephrosis	2023	10.3390/diagnostics13020247

Liu G	et al.	Comparative transcriptome analysis of miRNA in hydronephrosis male children caused by ureteropelvic junction obstruction with or without renal functional injury	2022	10.7717/peerj.12962
Liu J	et al.	Urinary microprotein concentrations in the long-term follow-up of dilating vesicoureteral reflux patients who underwent medical or surgical treatment	2016	10.1007/s11255-015-1097-7
Madsen MG	et al.	Epidermal growth factor and monocyte chemotactic peptide-1: Potential biomarkers of urinary tract obstruction in children with hydronephrosis	2013	10.1016/j.jpurol.2012.11.011
Madsen MG	et al.	Urinary NGAL, cystatin C, β2-microglobulin, and osteopontin significance in hydronephrotic children	2012	10.1007/s00467-012-2217-6
Mahyar A	et al.	The association of hypercalciuria and hyperuricosuria with vesicoureteral reflux in children	2017	10.1007/s10157-016-1236-1
Mahyar A	et al.	Serum interleukin -8 is not a reliable marker for prediction of vesicoureteral reflux in children with febrile urinary tract infection	2015	10.1590/S1677-5538.IBJU.2014.0381
Mandelia A	et al.	The role of urinary TGF-β_1, TNF-α, IL-6 and microalbuminuria for monitoring therapy in posterior urethral valves	2013	10.1007/s00467-013-2506-8
Mansilla M	et al.	Targeted broad-based genetic testing by next-generation sequencing informs diagnosis and facilitates management in patients with kidney diseases	2021	10.1093/ndt/gfz173
Mari A	et al.	Diagnostic Test Characteristics of Ultrasound Based Hydronephrosis in Identifying Low Kidney Function in Young Patients with Spina Bifida: A Retrospective Cohort Study	2021	10.1097/JU.0000000000001411
McLeod D	et al.	Common clinical markers predict end-stage renal disease in children with obstructive uropathy	2019	10.1007/s00467-018-4107-z
Mello MF	et al.	Evaluating TIMP-2 and IGFBP-7 as a predictive tool for kidney injury in ureteropelvic junction obstruction	2022	10.1590/S1677-5538.IBJU.2021.0340
Merrikhi A	et al.	Association of urinary transforming growth factor-β1 with the ureteropelvic junction obstruction	2014	10.4103/2277-9175.133196
Mittal PG	et al.	Correlation of urinary biomarkers (Interleukin-6, Transforming growth factor-β, E-Cadherin, and MCP-1) with conventional parameters of disease progression in patients of posterior urethral valves: A comparative analysis	2022	10.4103/jiaps.jiaps-182-21
Mohajerzadeh L	et al.	Value of Urine Neutrophil Gelatinase Associated Lipocalin for Prediction of Ureteropelvic Junction Obstruction in Children	2024	10.5812/numonthly-145156
Mohammadjafari H	et al.	Role of urinary levels of endothelin-1, monocyte chemotactic peptide-1, and N-Acetyl glucosaminidase in predicting the severity of obstruction in hydronephrotic neonates	2014	10.4111/kju.2014.55.10.670
Mohammadjafari H	et al.	The role of urinary TIMP1 and MMP9 levels in predicting vesicoureteral reflux in neonates with antenatal hydronephrosis	2014	10.1007/s00467-013-2693-3
Morozova OL	et al.	Urinary biomarkers of latent inflammation and fibrosis in children with vesicoureteral reflux	2020	10.1007/s11255-019-02357-1
Naik PB	et al.	Utility of urinary biomarkers neutrophil gelatinase-associated lipocalin and kidney injury molecule-1 as a marker for diagnosing the presence of renal scar in children with vesicoureteral reflux (VUR): A cross-sectional study	2022	10.4103/jiaps.JIAPS_334_20
Nickavar A	et al.	Utility of Urine Interleukines in Children with Vesicoureteral Reflux and Renal Parenchymal Damage	2021	10.22037/uj.v16i7.5957
Nickavar A	et al.	Validity of urine neutrophile gelatinase-associated lipocalin in children with primary vesicoureteral reflux	2020	10.1007/s11255-019-02355-3
Oktar T	et al.	Urinary HSP70 can predict the indication of surgery in unilateral ureteropelvic junction obstruction	2022	10.1007/s00383-021-05059-x
Otero HJ	et al.	DTI of the kidney in children: comparison between normal kidneys and those with ureteropelvic junction (UPJ) obstruction	2020	10.1007/s10334-019-00812-9
Panigrahi P	et al.	Role of Urinary Transforming Growth Factor Beta-B1 and Monocyte Chemotactic Protein-1 as Prognostic Biomarkers in Posterior Urethral Valve	2020	10.4103/jiaps.JIAPS_104_19

Park K	et al.	Role of urinary N-acetyl-beta-D-glucosaminidase in predicting the prognosis of antenatal hydronephrosis	2024	10.4111/icu.20240091
Parmaksız G	et al.	Role of new biomarkers for predicting renal scarring in vesicoureteral reflux: NGAL, KIM-1, and L-FABP	2016	10.1007/s00467-015-3194-3
Pastore V	et al.	Urinary excretion of EGF and MCP-1 in children with vesicoureteral reflux	2017	10.1590/S1677-5538.IBJU.2015.0132
Pavlaki A	et al.	The role of urinary NGAL and serum cystatin C in assessing the severity of ureteropelvic junction obstruction in infants	2020	10.1007/s00467-019-04349-w
Pizzini C	et al.	Urinary biomarkers in children with urinary tract infections with and without reflux on antibacterial prophylaxis with cefaclor	1999	10.2165/00044011-199918060-00005
Qi Y	et al.	Glomerular filtration rate measured by 99mTc-DTPA renal dynamic imaging is significantly lower than that estimated by the CKD-EPI equation in horseshoe kidney patients	2016	
Qian S	et al.	Urinary kidney injury molecule-1: a novel biomarker to monitor renal function in patients with unilateral ureteral obstruction	2020	10.1007/s11255-020-02528-5
Qin SL	et al.	Evaluation of matrix metalloproteinase-7 and matrix metalloproteinase-9 as biomarkers in ureteropelvic junction obstruction in children: A preliminary report	2021	10.12260/lcxewkzz.2021.07.013
Rathod KJ	et al.	Hydronephrosis due to pelviureteric junction narrowing: Utility of urinary enzymes to predict the need for surgical management and follow-up	2012	10.4103/0971-9261.91077
Rocha N	et al.	The protective arm of the renin–angiotensin system may counteract the intense inflammatory process in fetuses with posterior urethral valves	2019	10.1016/j.jped.2018.02.003
Saurabh N	et al.	Significance of inflammatory biomarkers and urethral histology in patients with urethral stricture disease in relation to treatment outcome—a single centre prospective study in the north-eastern part of India	2022	10.1186/s12301-021-00252-9
Scalabre A	et al.	Early detection of ureteropelvic junction obstruction in neonates with prenatal diagnosis of renal pelvis dilatation using (1)H NMR urinary metabolomics	2022	10.1038/s41598-022-17664-4
Sharma NK	et al.	Role of Urinary Biomarkers (Transforming Growth Factor β1, Neutrophil Gelatinase-Associated Lipocalin, and Cystatin C) as a Prognostic Factor of Renal Outcome in the Posterior Urethral Valve	2024	10.4103/jiaps.jiaps_210_23
Shirazi M	et al.	Evaluation of caspase 3 enzyme and TNF-alpha as biomarkers in ureteropelvic junction obstruction in children- a preliminary report	2017	10.12669/pjms.332.11934
Spaggiari E	et al.	Sequential fetal serum β2-microglobulin to predict postnatal renal function in bilateral or low urinary tract obstruction	2017	10.1002/uog.15968
Suchiang B	et al.	Role of urinary Neutrophil Gelatinase-Associated Lipocalin (NGAL), Monocyte Chemoattractant Protein-1(MCP-1), and Interleukin-6(IL-6) as biomarkers in pediatric patients with hydronephrosis	2022	10.1007/s00383-022-05207-x
Taranta-Janusz K	et al.	New tubular injury markers in children with a solitary functioning kidney	2014	10.1007/s00467-014-2802-y
Tokuc E	et al.	Inflammation indexes and machine-learning algorithm in predicting urethroplasty success	2024	10.4111/icu.20230302
Tomotaki S	et al.	Association between cord blood cystatin C levels and early mortality of neonates with congenital abnormalities of the kidney and urinary tract: a single-center, retrospective cohort study	2017	10.1007/s00467-017-3733-1
Topaktaş R	et al.	Hematologic parameters and Neutrophil / Lymphocyte ratio in the prediction of urethroplasty success	2019	10.1590/S1677-5538.IBJU.2018.0682
Trnka P	et al.	Urinary biomarkers in obstructive nephropathy	2012	10.2215/CJN.09640911
Turczyn A	et al.	Serum and urine periostin and cytokeratin-18 in children with congenital obstructive nephropathy	2022	10.5114/CEJI.2022.115687

Vasconcelos MA	et al.	Urinary levels of TGF β-1 and of cytokines in patients with prenatally detected nephrouropathies	2011	10.1007/s00467-011-1802-4
Vieira É	et al.	Posterior urethral valve in fetuses: evidence for the role of inflammatory molecules	2017	10.1007/s00467-017-3614-7
Wang HS	et al.	Association between urinary biomarkers MMP-7/TIMP-2 and reduced renal function in children with ureteropelvic junction obstruction	2022	10.1371/journal.pone.0270018
Wang Q	et al.	Matrix Remodeling-Associated Protein 5 in Urinary Exosomes as a Potential Novel Marker of Obstructive Nephropathy in Children With Ureteropelvic Junction Obstruction	2020	10.3389/fped.2020.00504
Washino S	et al.	A novel biomarker for acute kidney injury, vanin-1, for obstructive nephropathy: A prospective cohort pilot study	2019	10.3390/ijms20040899
Wasilewska A	et al.	KIM-1 and NGAL: New markers of obstructive nephropathy	2011	10.1007/s00467-011-1773-5
Xie J	et al.	The relationship between amniotic fluid miRNAs and congenital obstructive nephropathy	2017	
Xuan X	et al.	Plasma MCP-1 and TGF-β1 Levels are Associated with Kidney Injury in Children with Congenital Anomalies of the Kidney and Urinary Tract	2024	10.1007/s12010-023-04808-z
Yeh HM	et al.	Biomarkers and echocardiography for evaluating the improvement of the ventricular diastolic function after surgical relief of hydronephrosis	2017	10.1371/journal.pone.0188597
Yousefichaijan P	et al.	The effect of vitamin A on clinical manifestations of recurrent pyelonephritis in children	2020	10.5812/numonthly.103278
Zeybek SG	et al.	Can urinary caspase-3 and cytochrome c levels be used as predictive biomarkers in the management of unilateral antenatal hydronephrosis?	2024	10.1007/s11255-024-04008-6
Zhao Q	et al.	ATP5B and ETFB metabolic markers in children with congenital hydronephrosis	2016	10.3892/mmr.2016.5914
Zheng Q	et al.	Computer-aided diagnosis of congenital abnormalities of the kidney and urinary tract in children based on ultrasound imaging data by integrating texture image features and deep transfer learning image features	2019	10.1016/j.jpurol.2018.10.020
